# TFPAG-Net: A Time-Frequency Dual-Branch Fusion and PCMCI-Based Association-Guided Network for IIoT Intrusion Detection

**DOI:** 10.3390/s26185865

**Published:** 2026-09-16

**Authors:** Haoran Lei, Shiming Li, Wentao Li, Shenglin Wang, Yuntao Ni

**Affiliations:** College of Computer Science and Information Engineering, Harbin Normal University, Harbin 150025, China; hranlei@163.com (H.L.); wentaolis@163.com (W.L.); wangshenglins@163.com (S.W.)

**Keywords:** Industrial Internet of Things, intrusion detection, time–frequency feature fusion, multi-scale temporal pyramid, association guidance, lightweight

## Abstract

The Industrial Internet of Things (IIoT) is being used a lot in important areas like advanced manufacturing, smart energy systems, and intelligent cities. Watching for intrusion detection is very important for the safety of the IIoT. Nevertheless, multivariate sensor sequences frequently demonstrate pronounced physical coupling, non-stationarity, and periodicity concurrently, rendering it prone for detection models grounded in statistical correlation to erroneously classify normal collaborative variations as anomalies. Moreover, prevailing methods predominantly concentrate on single-domain representations within either the time or frequency domain, posing challenges in addressing both burst and periodic attacks concurrently. To address these challenges, this article introduces the Time–Frequency Dual-Branch Fusion and PCMCI-Based Association-Guided Network (TFPAG-Net) for IIoT Intrusion Detection. This model initially constructs a lightweight temporal convolutional network backbone employing depthwise separable convolutions. Subsequently, parallel branches in the time and frequency domains are established to respectively model local abrupt changes, long-range dependencies, and periodic spectral structures, with time–frequency feature fusion facilitated through a sample-dependent gating mechanism. Building on this, multi-scale temporal pyramids are employed to amalgamate fine, intermediate, and coarse-scale information. Furthermore, as an auxiliary refinement, a lagged conditional-dependence prior estimated from the training data via PCMCI is projected into a bounded attention bias to provide supplementary guidance for channel feature reweighting. Evaluations on Edge-IIoTset, X-IIoTID, and SWaT yield mean Macro-F1 scores of 0.9886, 0.9466, and 0.9607, respectively, over five predefined random seeds. Under the unified training protocol, TFPAG-Net ranks second on Edge-IIoTset and achieves the highest mean Macro-F1 on X-IIoTID and SWaT. Ablation experiments show dataset-dependent effects of the proposed components. On Edge-IIoTset and X-IIoTID, the final attention-stage improvement reflects the joint effect of SE-based modulation and the PCMCI-derived association prior. Accordingly, PCMCI is treated as an auxiliary association refinement rather than a principal contribution of TFPAG-Net. Additionally, TFPAG-Net maintains a moderate computational footprint, with approximately 0.29 M parameters, providing a favorable balance between model complexity and intrusion detection performance.

## 1. Introduction

The Industrial Internet of Things (IIoT) serves as a pivotal technological foundation for advancing the digitization, networking, and intelligence of production systems within the framework of Industry 4.0. By integrating Programmable Logic Controllers, Remote Terminal Units, industrial sensors, and actuators into communication networks, IIoT facilitates device status awareness, production process monitoring, and the issuance of control commands, thereby substantially elevating the automation level, production efficiency, and resource utilization of industrial systems [1,2].

Nevertheless, the transition of industrial systems from a relatively enclosed operational environment to an open, interconnected network has markedly broadened their cyberattack surface [3,4]. In contrast to traditional information networks, IIoT is characterized by weak protocol security mechanisms, constrained device resources, stringent real-time requirements, and tangible physical consequences of attacks. Industrial protocols such as Modbus TCP and DNP3 were primarily designed for trusted environments and exhibit deficiencies in identity authentication, data encryption, and access control [5]. Attackers may disrupt industrial processes by forging control instructions. Moreover, industrial sensor nodes and edge gateways are frequently constrained by computational power, storage capacity, and energy consumption, rendering the deployment of intricate and computationally intensive security detection models challenging [6,7]. Concurrently, industrial control systems impose stringent latency constraints, necessitating that the intrusion detection process does not significantly impede normal production and control tasks. More critically, IIoT attacks have the potential to propagate from the information space to the physical space, resulting in equipment damage, production halts, and even safety incidents. Therefore, it is of paramount importance to investigate IIoT intrusion detection methods that integrate accuracy, real-time performance, and lightweight characteristics.

An Intrusion Detection System (IDS) constitutes a vital technical approach to safeguarding industrial network security by monitoring network traffic, device status, and system logs, identifying potential malicious activities and triggering alerts [8]. Traditional IDS based on feature signatures rely on pre-established attack rule libraries, which are effective in detecting known attacks yet exhibit limited capabilities in identifying attack variants and zero-day attacks. In contrast, anomaly-based IDS learn data distributions and behavioral patterns under normal operating conditions to detect anomalies that deviate from the established baseline, thereby possessing the potential to identify unknown attacks. In recent years, deep learning, with its proficiency in automated feature extraction and modeling of intricate nonlinear relationships, has been extensively employed in industrial network intrusion detection and has achieved commendable detection outcomes [9,10].

Despite the advancements made by existing deep learning IDS, they still confront the following two vital challenges within the context of IIoT sensor networks. Firstly, the statistical correlation between sensor variables struggles to accurately reflect their structural dependencies. Variables such as temperature, pressure, flow rate, and liquid level in industrial processes often exhibit synchronous changes due to control logic, equipment interconnections, and shared external factors; however, correlation does not imply direct dependency or causality. Traditional deep learning models predominantly learn statistical patterns and lack explicit constraints on time-lag relationships and industrial structures, which may hinder their ability to identify attacks that contravene physical laws despite normal statistical distributions or misconstrue normal coupling as anomalies, leading to false alarms and alarm fatigue [11,12,13]. Secondly, a singular feature domain is inadequate to comprehensively characterize IIoT attacks. Denial of service, scanning probes, and burst injections typically manifest as localized mutations or pulses in the time domain, whereas periodic injections, low-rate attacks, and covert control interferences may generate abnormal peaks or energy shifts in the frequency domain. Existing methods often concentrate on single-domain modeling and lack a time–frequency coordination mechanism, making it arduous to encompass diverse attack patterns [14,15]. It should also be noted that IIoT security encompasses complementary system-level problems, including device identification, network-level anomaly detection, and interactions among heterogeneous system components. The scope of this study is more specific: it focuses on multivariate sensor, network, and control sequences and investigates how complementary temporal–spectral representations and training-derived inter-variable association information can be integrated for intrusion classification.

To address the above challenges, this study proposes the Time–Frequency Dual-Branch Fusion and PCMCI-Based Association-Guided Network for IIoT intrusion detection. The model first employs depthwise-separable dilated convolutions to construct a lightweight TCN backbone. Parallel temporal and frequency–domain branches are then used to learn complementary representations: the temporal branch preserves temporal ordering and event localization information, whereas the frequency–domain branch emphasizes spectral amplitude and periodic patterns. Because the Fourier phase is discarded in the frequency branch, its output is treated as a learned spectral embedding rather than a reconstruction of the original temporal signal. The two branch representations are combined through a sample-dependent gating mechanism and subsequently aggregated by a multi-scale temporal pyramid. As an auxiliary refinement, PCMCI is applied exclusively to the training partition to estimate lagged conditional-dependence statistics among the original variables. The retained association information is compressed into a lightweight variable level prior and projected into the latent channel attention space as a bounded soft bias. This prior provides supplementary dataset-level association information for feature reweighting rather than serving as a principal architectural component or establishing a validated physical causal graph.

The main contributions of this paper are summarized as follows.

1. Propose a time–frequency dual-branch collaborative modeling architecture. To address the problem that different IIoT attacks may exhibit distinct characteristics in the temporal and frequency domains, parallel time–domain and frequency–domain branches are constructed based on the shared TCN features. The time–domain branch focuses on local mutations and temporal dependencies, while the frequency–domain branch captures spectral amplitude and periodic patterns. A sample-dependent gating mechanism is further introduced to dynamically combine the two types of features according to different input samples, thereby improving the model’s ability to represent heterogeneous attack patterns.
2. Design a multi-scale temporal aggregation mechanism for fine-to-coarse contextual modeling. The gated time–frequency representation is aggregated at multiple temporal resolutions to capture fine, intermediate, and coarse-scale information, thereby enhancing the utilization of heterogeneous temporal patterns. A training-derived lagged conditional dependence prior is additionally incorporated as an auxiliary bounded bias in the subsequent channel attention module to provide supplementary variable association guidance; it is not treated as an independent principal contribution.
3. Build a lightweight TCN feature extraction backbone. Depthwise separable convolution is combined with dilated convolution to enlarge the temporal receptive field while reducing the number of parameters and computational overhead. On this basis, the temporal backbone, time–frequency dual branches, multi-scale aggregation, and association-guided attention are integrated into a unified intrusion detection framework, providing a relatively lightweight implementation for IIoT intrusion detection.

The ensuing sections of this paper are organized as follows: Section 2 introduces related work; Section 3 describes the overall framework of the model and the specific implementation of the methods used; Section 4 presents specific experiments and analysis; lastly, Section 5 provides a summary of the research presented in this paper and draws pertinent conclusions.

## 2. Related Work

### 2.1. Deep Intrusion Detection

Deep learning, leveraging its robust capability for automatic feature extraction, has emerged as the predominant technical paradigm in intrusion detection, evolving alongside advancements in model designs.

Hochreiter et al. recommended the Long Short-Term Memory (LSTM) network [16], which effectively captures long-term temporal dependencies through its gating mechanisms and is extensively employed in temporal anomaly detection. Within the background of IIoT intrusion detection, Ben Said et al. [17] proposed a CNN-BiLSTM hybrid model that initially extracts local spatial features using a one-dimensional convolutional layer and subsequently captures bidirectional temporal dependencies through a bidirectional LSTM. This model demonstrated exceptional results on the UNSW-NB15 and NSL-KDD datasets. Nevertheless, RNN-based models are prone to the vanishing/exploding gradient problem and lack parallelization capabilities, resulting in reduced training efficiency.

CNNs excel at efficiently extracting local features through local receptive fields and weight-sharing mechanisms. In temporal data processing, one-dimensional CNNs (Conv1D) can directly process temporal signals. The FFT-RDNet model proposed by Xiang et al. [18] employs depthwise separable convolutions [19] and frequency domain feature fusion to achieve lightweight IoT intrusion detection, maintaining high detection performance while minimizing parameter count.

The Transformer design helps to understand relationships over long distances using a self-attention method. It has also been changed for analyzing time series data after doing very well in natural language processing (NLP) and computer vision (CV). Zhang et al. [20] integrated the self-attention mechanism of Transformer with the local perception capabilities of CNN-BiLSTM, proposing a hybrid IDS framework that surpasses single-architecture models across multiple benchmark datasets [21,22]. However, the computational complexity of the self-attention mechanism in Transformer model quadratic scaling with respect to the sequence length (O(L^2^)) imposes substantial computational overhead on long-term IIoT data.

TCN utilizes a causal dilated convolution structure where the receptive field expands exponentially with network depth, enabling it to catch long-range temporal dependencies while supporting parallel computation [23]. Compared with recurrent architectures, TCNs avoid sequential state propagation, while their computational cost scales approximately linearly with sequence length when the kernel size and channel configuration are fixed. In this article, TCN is adopted as the backbone network, with depthwise separable convolutions incorporated to further reduce parameter count.

TimesNet, proposed by Wu et al. [24], converts one-dimensional time series into two-dimensional tensors through Fast Fourier Transform (FFT) and employs the Inception module to simultaneously capture intra-period and inter-period variation patterns, achieving outstanding performance across various time series analysis tasks. The fundamental insight of this approach is that the periodicity of time series allows their representation as the superposition of multiple periodic components in the frequency domain, enabling simultaneous modeling of intra-period and inter-period dependencies through two-dimensional convolution.

Beyond sensor-level sequence modeling, recent IoT security research has also considered complementary system-level perspectives. Rabbani et al. [25] jointly investigated IoT device identification and anomaly detection by integrating packet-based and flow-based features in heterogeneous IoT environments. In another direction, Cimino and Deufemia [26] modeled interactions among trigger action rules through scene interaction graphs to detect potentially unsafe interference in IoT automation systems. These studies highlight the importance of device heterogeneity and system component interactions in IoT security. In contrast, the scope of the present work is multivariate IIoT sensor, network, and control sequences with the objective of learning complementary temporal–spectral representations and exploiting training-derived inter-variable association information for intrusion classification.

Recent reviews of learning-based IoT/IIoT intrusion detection [8,9,10] indicate that existing approaches typically emphasize particular aspects of the detection problem, including temporal sequence modeling, frequency or time–frequency representation, lightweight feature extraction, or relational structure modeling. Relatively fewer approaches jointly coordinate explicit time–domain and frequency–domain representations with lightweight inter-variable structural guidance. Accordingly, the contribution of TFPAG-Net lies not in introducing these individual techniques independently but in integrating a lightweight temporal backbone, parallel temporal–spectral representation learning, sample-dependent gated fusion, multi-scale temporal aggregation, and association-guided feature reweighting within a unified IIoT intrusion detection framework.

### 2.2. Application of Time–Frequency Analysis in Anomaly Detection

Time–frequency analysis, by preserving both time and frequency information concurrently, offers a comprehensive depiction of the time-varying spectral characteristics of signals, thereby attracting considerable attention in the realm of time series anomaly detection. The Fourier transform changes signals from the time they happen into their frequency parts, making it easier to see the different frequencies in the signal. Nevertheless, the conventional Fourier transform presupposes signal stationarity and is incapable of delineating temporal variations in frequency. The Short-Time Fourier Transform (STFT) locally applies the Fourier transform through windowing techniques, thereby providing time–frequency localization information. However, its performance is constrained by a fixed window length, as dictated by the Heisenberg uncertainty principle [27]. Within the domain of intrusion detection, FFT-RDNet leverages FFT on IoT traffic characteristics, extracting frequency–domain energy distributions as the foundation for anomaly detection [18].

Wavelet transform, through scaling and translation operations, facilitates multi-resolution time–frequency analysis and finds extensive application in time series anomaly detection. Wavelet time–frequency analysis has proven effective in capturing periodic attack patterns in malicious traffic detection [28]. Its multi-scale attributes enable the detection of both transient pulse-type attacks and prolonged periodic attacks. However, wavelet transform is computationally intensive, and the selection of wavelet bases significantly influences performance, with no universally applicable selection criterion available. The Wigner–Ville distribution offers a high-resolution time–frequency representation, however, which is plagued by cross-term obstruction and exhibits high computational complexity in multidimensional signal processing [29].

Lately, researchers have started looking into using deep learning to automatically obtain time–frequency information. FreqWave-TranDuD [28] integrates Fourier transform with wavelet transform, employing an encoder–decoder architecture to extract time–frequency features for multivariate time series abnormality detection. Recent studies have further explored antinoise adaptive time–frequency fusion [30], frequency-enhanced Transformer decomposition [31], and frequency–domain restoration combined with dynamic channel graph sparsification [32], further demonstrating the value of frequency-aware representations for multivariate anomaly detection. This approach validly harnesses the global frequency information of Fourier transform and the multi-resolution localization capabilities of wavelet transform while maintaining computational efficiency.

These studies demonstrate that spectral and time–frequency representations can provide information that is complementary to purely temporal modeling. In particular, periodic or slowly varying abnormal behaviors may produce characteristic changes in spectral-energy distributions even when their point-wise temporal deviations are relatively weak. Conversely, abrupt attacks and transient disturbances generally retain more explicit localization information in the time domain. Existing approaches therefore provide strong evidence for the usefulness of frequency-enhanced representations; however, their processing strategies differ in how temporal and spectral information is constructed and combined.

### 2.3. Temporal Association Learning and Causal Discovery

Causal discovery and temporal dependency analysis provide structured approaches for investigating relationships among variables in multivariate time series. However, statistical dependence, temporal directionality, and physical causality represent distinct concepts. A directed or lagged relationship identified from observational data should therefore not automatically be interpreted as evidence of a physical causal mechanism. Nevertheless, conditional dependence and temporal association structures can provide informative priors for multivariate anomaly and intrusion detection when they are interpreted under appropriate assumptions.

The PC algorithm [33] stands as the most quintessential constraint-based causal discovery method, discerning the causal structure between variables via iterative conditional independence tests. Nevertheless, the computational complexity of the PC algorithm escalates exponentially in high-dimensional contexts, rendering its application challenging for IIoT datasets with elevated dimensions. The PCMCI algorithm, proposed by Runge et al. [34], markedly diminishes computational complexity through a two-stage approach (PC conditional screening plus MCI test), enabling scalable causal inference discovery within high-dimensional time-series data. Runge [35] further advanced the field by proposing Conditional Mutual Information Estimation based on k-Nearest Neighbors (CMIknn), which bolsters nonlinear causality testing and augments the applicability of PCMCI in nonlinear settings. Debeire et al. [36] subsequently introduced bootstrap aggregation and confidence measures to improve the robustness of time-series causal discovery.

In addition to constraint-based methods, score-based approaches search for graph structures by optimizing a predefined scoring criterion. Such methods provide flexible alternatives for structure learning but may incur considerable computational costs and can be affected by the optimization landscape. More recent studies have introduced machine-learning-based strategies for causal discovery. For example, Zhu et al. [37] employed reinforcement learning to search over causal graph structures, whereas Shen et al. [38] combined graph search with a neural conditional-dependence measure. These methods improve the flexibility of structure learning but generally introduce additional optimization and computational requirements.

Structural information has also been explored for multivariate anomaly detection. Zhang et al. [12] detected anomalies in industrial processes by characterizing changes in graph structures among multivariate variables, while Heppel et al. [13] investigated the use of causal information for multivariate time-series anomaly detection. Liu et al. [39] proposed GCAD, which detects multivariate anomalies from changes in Granger causal predictive relationships. These studies demonstrate that dependency structures can provide useful information beyond conventional point-wise feature representations, although the interpretation and utilization of the learned structures differ substantially across methods.

The approach adopted in TFPAG-Net differs from methods that directly perform graph message passing or treat an estimated dependency graph as a complete causal representation. PCMCI is applied only to the training partition to estimate directed lagged conditional-dependence statistics, which are subsequently compressed into a lightweight variable-level prior and projected into the latent channel attention space. The use of PCMCI in TFPAG-Net is motivated by previous comparative studies in other multivariate time-series domains. Docquier et al. [40] reported that PCMCI and related causal discovery approaches were effective in reducing spurious links compared with conventional correlation analysis. Similarly, Yadav et al. [41] showed that multivariate causal discovery methods, including PCMCI+, produced fewer false-positive drivers than Pearson correlation in complex hydrometeorological systems, while Pearson correlation and Spearman’s rho primarily characterize statistical association. These findings provide methodological motivation for adopting a conditional-dependence-based estimator rather than relying solely on pairwise correlation. However, because these results originate from other application domains, they are not used here to establish the superiority of PCMCI in IIoT intrusion detection. Accordingly, PCMCI is employed only as an auxiliary-training-derived association prior rather than a standalone methodological contribution.

## 3. Model Structure

### 3.1. Overall Framework

**Figure 1:**
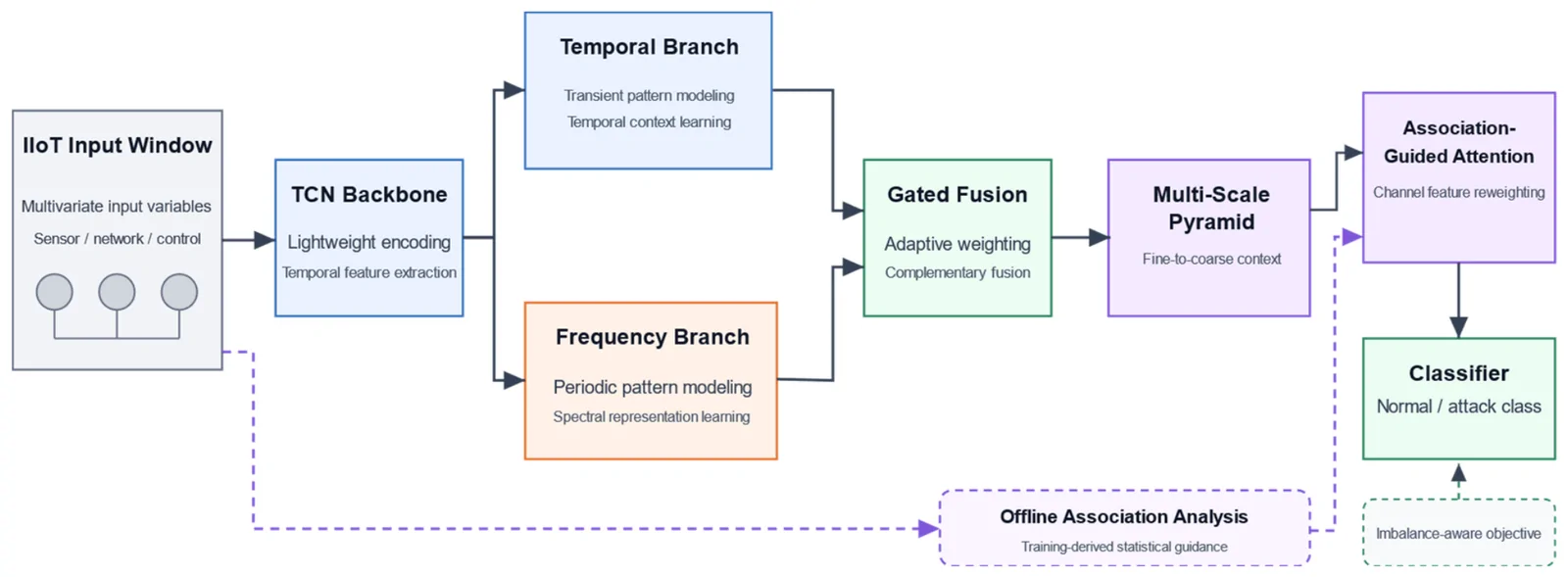
TFPAG-Net overall framework. Solid arrows indicate the main forward feature flow; purple dashed arrows indicate the offline association-guidance path, and the green dashed arrow indicates the training objective. Different colors distinguish the functional modules.

Motivated by the limited computation and memory budgets commonly encountered in IIoT gateways, TFPAG-Net is designed to control the parameter growth introduced by multi-branch representation learning while modeling heterogeneous attack patterns and inter-variable associations. The overall architecture of the model is illustrated in Figure 1. To tackle the challenge of inadequately characterizing both abrupt and periodic attack patterns within a single feature domain, TFPAG-Net establishes parallel time–domain and frequency–domain branches. These branches are designed to model temporal transitions and event localization information, as well as periodic and spectral-amplitude patterns, from complementary representational perspectives. Leveraging the joint features extracted by the two branches, the model generates sample-dependent gated weights to facilitate adaptive fusion of temporal and spectral representations. Subsequently, the fused features are aggregated at multiple resolutions to capture fine, intermediate, and coarse-scale contextual information. Concurrently, PCMCI is applied exclusively to the training partition to estimate lagged conditional-dependence statistics among the original input variables. The retained associations are first summarized into a directed source target representation and are then compressed into a lightweight variable-level association prior, with target-specific and exact lag-specific information no longer retained after this compression. This prior is projected into the latent channel attention space and bounded through a tanh transformation before being added to the sample-dependent channel attention logits. The final sigmoid operation produces bounded channel weights, which softly reweight the multi-scale representation before pooling and classification. In this manner, the PCMCI-derived information is incorporated as an observational association prior rather than as a hard structural constraint or evidence of physical causality, enabling the network to exploit complementary time–frequency information while introducing lightweight statistical guidance into the learned representation.

### 3.2. TCN Backbone Network

**Figure 2:**
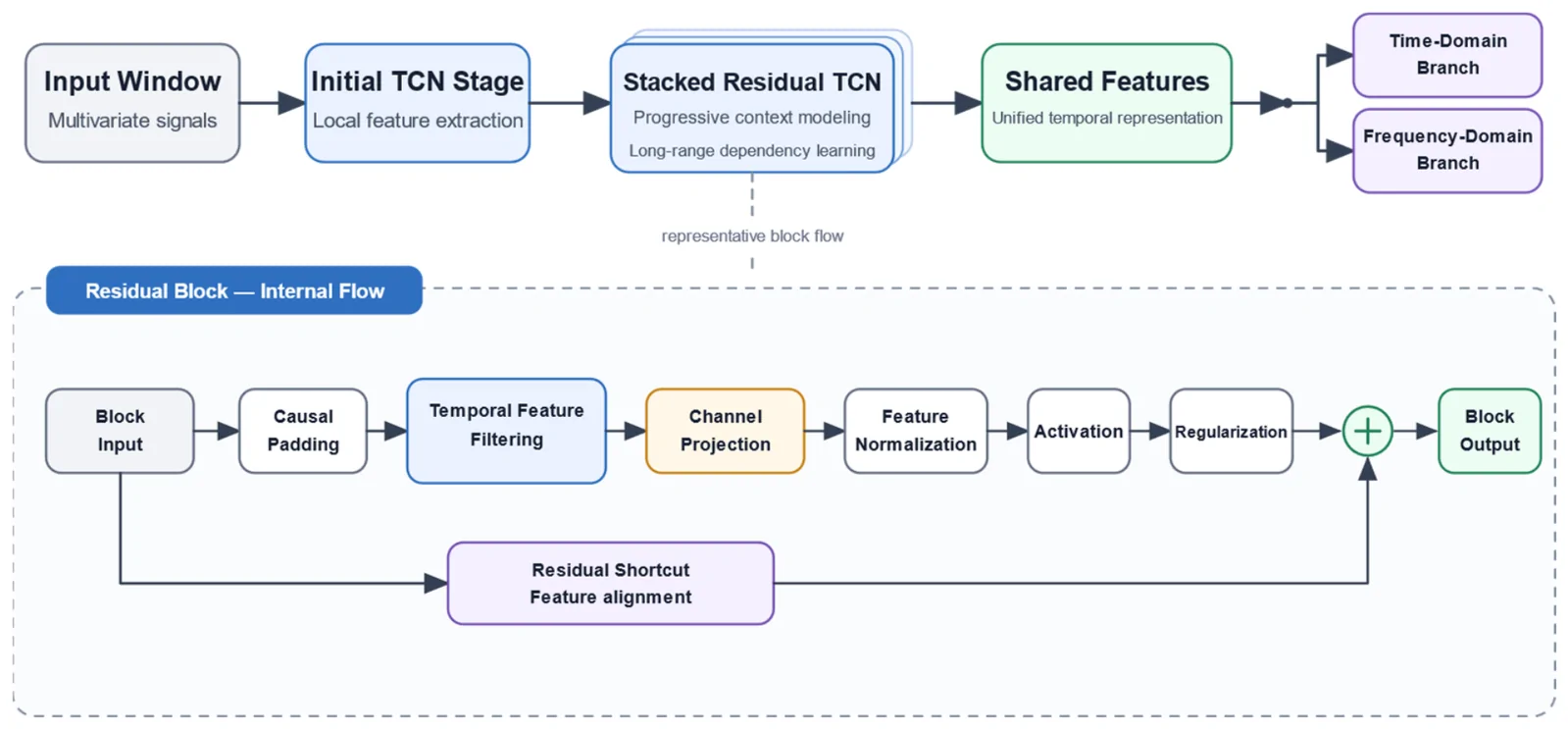
Lightweight TCN backbone network structure diagram. The dashed box illustrates the internal flow of a residual block; arrows indicate feature propagation, the “+” symbol denotes residual addition, and different colors distinguish functional operations and stages.

This study focuses on effectively capturing long-term temporal dependencies in multivariate IIoT time-series data while controlling parameter and computational overhead. To achieve this goal, a Temporal Convolutional Network (TCN) [23] is employed as the shared temporal backbone for feature extraction, as illustrated in Figure 2. By introducing dilated convolutions, TCN progressively enlarges the temporal receptive field without requiring recurrent computation, thereby enabling parallel processing across sequence positions and improving the efficiency of temporal feature modeling. To further reduce the number of parameters and computational overhead, the conventional one-dimensional convolution is replaced with depthwise separable dilated convolution, allowing the backbone to preserve temporal modeling capability while maintaining a lightweight architecture.

It should be noted that the term causal convolution used in this section specifically refers to an architectural operation that allows for the computation of features at the current time step without utilizing samples from future positions within the convolutional receptive field. This architectural notion of causality is fundamentally different from the causal interpretation associated with PCMCI discussed in Section 3.6 and should not be understood as implying a physical or statistical causal relationship between IIoT variables.

**Figure 3:**
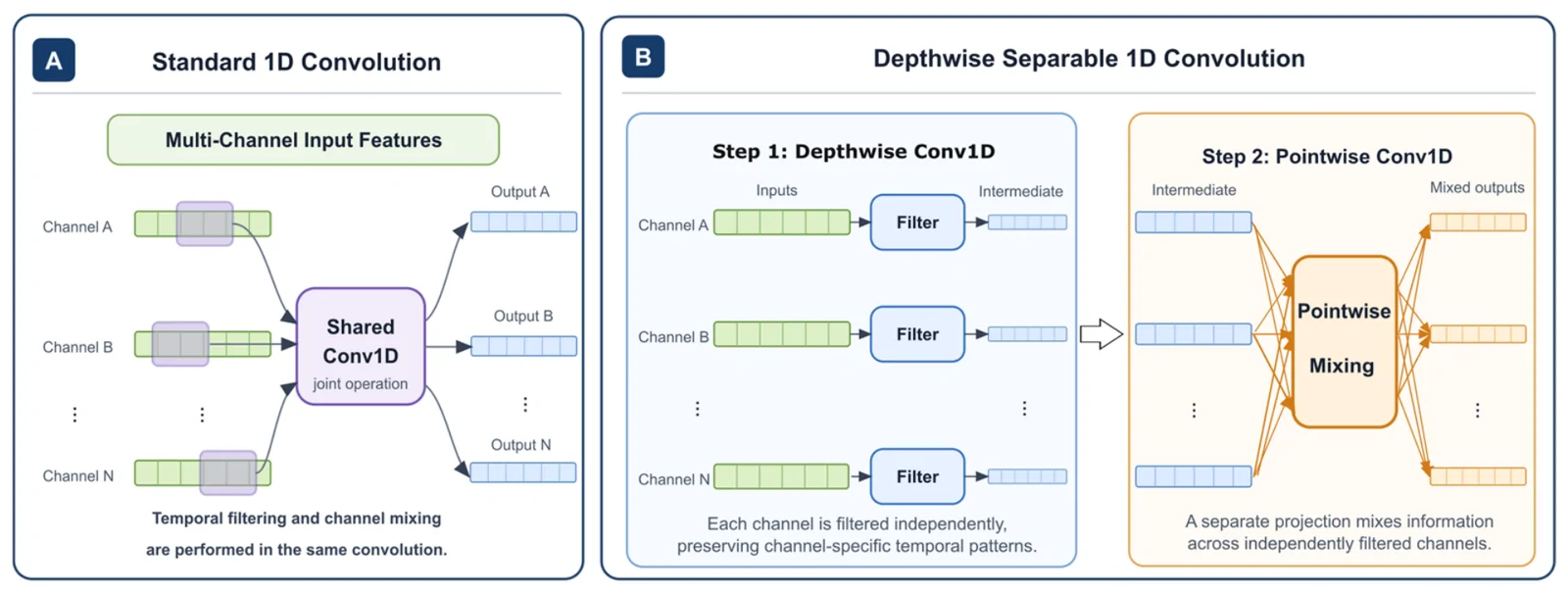
Comparison of structure and complexity between standard convolution and depthwise separable convolution. Panel A illustrates standard convolution, whereas Panel B illustrates depthwise separable convolution; different colors distinguish channel-wise filtering and pointwise channel mixing.

**1.**  **Depthwise Separable Dilated Convolution.** The computational complexity of a standard one-dimensional convolutional layer is $O(B\cdot L\cdot D_{in}\cdot D_{out}\cdot k)$, where B represents the batch size, L denotes the sequence length, $D_{in}$ and $D_{out}$ signify the numbers of input and output channels, respectively, and k stands for the size of the convolutional kernel. In order to reduce the number of parameters, this paper adopts depthwise separable convolution [19]. The structure and complexity comparison between standard convolution and depthwise separable convolution is illustrated in Figure 3. Depthwise separable convolution factorizes the standard convolutional operation into two successive steps:
  **(1)**  **Depthwise Convolution.** Perform a causal convolution operation with a length of k independently for each input channel, where the number of output channels equals the number of input channels. The computational complexity is $O(B\cdot L\cdot D_{in}\cdot k)$.
  **(2)**  **Pointwise Convolution.** 1×1 convolution was used to fuse the output of the channel-by-channel convolution, and the number of channels was adjusted to the target dimension. The computational complexity is $O(B\cdot L\cdot D_{in}\cdot D_{out})$. The total complexity of depthwise separable convolution is $O(B\cdot L\cdot D_{in}\cdot (k+D_{out}))$.

Compared with standard convolution, the ratio between their dominant multiplication costs is $\frac{B\cdot L\cdot D_{in}\cdot D_{out}\cdot k}{B\cdot L\cdot D_{in}\cdot (k+D_{out})}=\frac{k\cdot D_{out}}{k+D_{out}}$. When $D_{out}\gg k$, this ratio approaches k, indicating that depthwise separable convolution can substantially reduce the computational burden while retaining temporal filtering and cross-channel feature mixing as two explicit operations.

**2.**  **Dilated Causal Convolution.** In order to capture the long-distance temporal dependence, this paper introduces the dilation rate d based on the depthwise separable convolution.

Dilated convolution increases the temporal receptive field without proportionally increasing the number of parameters. Let the kernel size at each layer be k, and let $d_{i}$ denote the dilation factor of the i-th layer. The receptive field after l stacked convolutional layers is

(1) $${\mathrm{RF}}_{l}=1+\sum_{i=1}^{l}\left(k-1\right)\cdot d_{i}$$

For the exponentially increasing dilation schedule $d_{i}=2^{i-1}$, Equation (1) becomes

(2) $$RF_{l}=1+\left(k-1\right)\left(2^{l}-1\right)$$

The backbone in this study contains three depthwise-separable dilated convolutional layers with k=3 and d={1,2,4}, giving $RF_{3}=1+2\left(1+2+4\right)=15.$

Thus, each high-level backbone feature can directly integrate information from a 15-step temporal receptive field. This value describes the receptive field of the three-layer backbone itself and is not interpreted as a universal temporal range sufficient for all IIoT attack patterns; additional temporal processing is subsequently performed by the time–domain branch and multi-scale aggregation modules.

The channel configuration is as follows:

Layer 1: Conv1d(D→64,k=3,d=1)→Batch Normalization→ReLU→Dropout(0.1)
Layer 2: Conv1d(64→128,k=3,d=2)→Batch Normalization→ReLU→Dropout(0.1)
Layer 3: Conv1d(128→256,k=3,d=4)→Batch Normalization→ReLU→Dropout(0.1)

**3.**  **The Residual Causal Block.** To further improve the stability of network training and facilitate gradient propagation, each TCN layer is implemented as a residual causal block. As illustrated in Figure 2, the main transformation path sequentially performs left causal padding, depthwise dilated convolution, pointwise 1 × 1 convolution, Batch Normalization, ReLU activation, and Dropout. For the input feature $H^{\left(l-1\right)}$ of the l-th residual block, the output is expressed as
  (3) $$H^{\left(l\right)}=F\left(H^{\left(l-1\right)}\right)+S\left(H^{\left(l-1\right)}\right)$$

Batch Normalization stabilizes feature distributions and facilitates convergence, while ReLU enhances nonlinear representation and Dropout helps reduce overfitting. When the input and output channel dimensions are identical, an identity shortcut is used; otherwise, a convolution aligns the channel dimensions before element-wise addition. The residual connection provides a direct information and gradient propagation path, thereby improving optimization stability in the stacked TCN backbone [42].

### 3.3. Time–Frequency Dual Branch Structure

**Figure 4:**
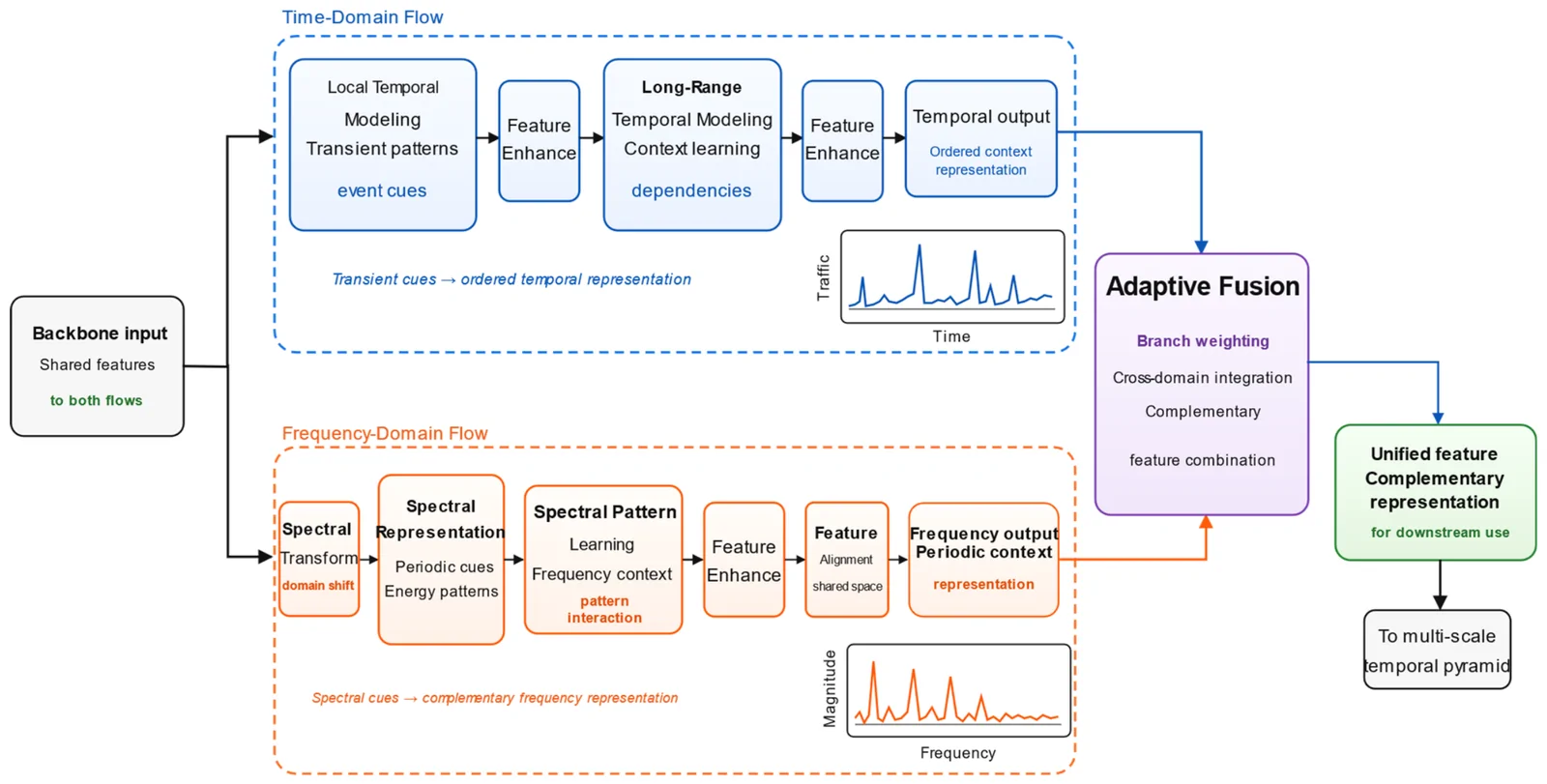
Architecture of the time–frequency dual-branch module. Blue and orange dashed boxes denote the time-domain and frequency-domain flows, respectively; solid arrows indicate feature flow, while the purple and green modules denote adaptive fusion and the unified output representation.

The time–frequency dual-branch structure is a core module of TFPAG-Net. As illustrated in Figure 4, both branches receive the same TCN backbone representation but learn different feature semantics. The time–domain branch retains temporal ordering and event localization information, whereas the frequency–domain branch emphasizes spectral amplitude structure and periodic patterns. By processing the shared backbone representation from complementary temporal and spectral perspectives, the two branches provide distinct latent representations that are subsequently combined by the gated fusion module. Their empirical contribution is dataset-dependent and evaluated through the experiments in Section 4.

Let the shared TCN backbone representation be denoted by $H_{tcn}\in R^{B\times C\times T}$, where B is the batch size, C=256 is the latent channel dimension, and T is the temporal length of the input window.

**Figure 5:**
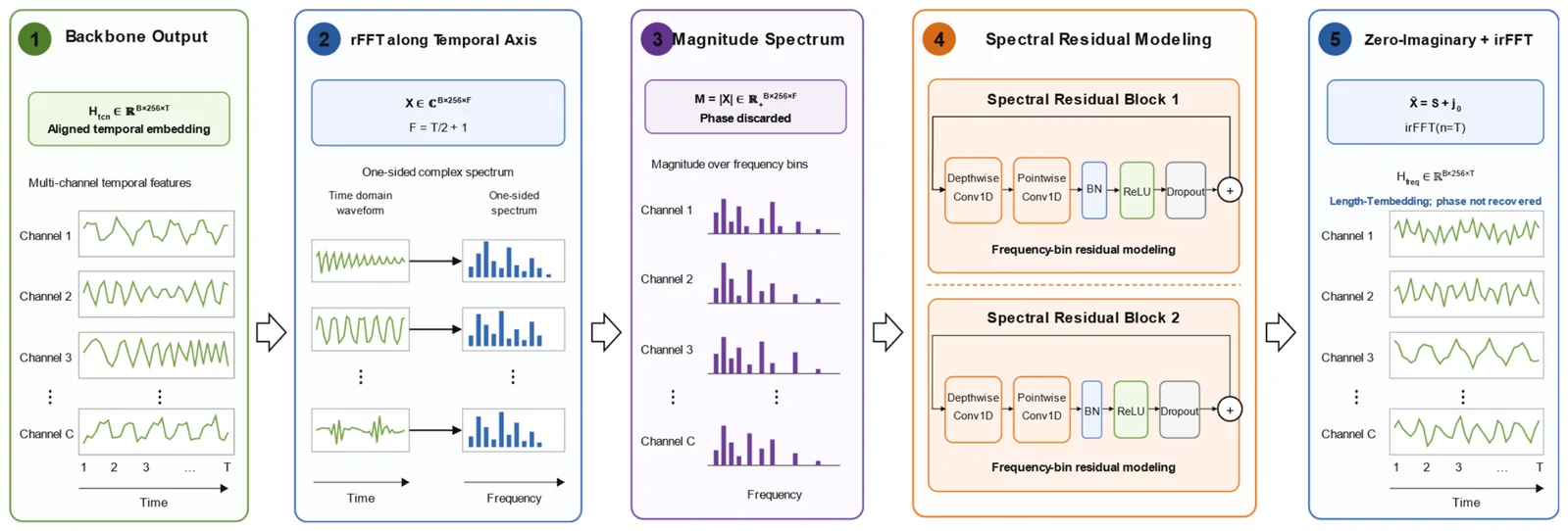
rFFT-based frequency–domain branch processing flow. Arrows indicate the sequential processing stages; different colors distinguish the five processing stages, and the “+” symbol denotes residual addition within the spectral residual blocks.

**1.**  **Time**–**Domain Branch.** The temporal branch stacks two residual TCN blocks on the backbone output $H_{tcn}$ to retain the complete temporal structure to further mine the local dynamic changes and long-term temporal dependencies in the time dimension. Causal padding preserves the temporal length, while the dilation schedule enlarges the effective temporal context. The output is denoted by $H_{time}\in R^{B\times 256\times T}$, which preserves the ordering of temporal positions and therefore provides event localization information within each input window. Accordingly, the temporal branch is designed to emphasize abrupt variations and temporally localized patterns within each input window.
**2.**  **Frequency**–**Domain Branch.** Some industrial network attacks show obvious periodicity or frequency characteristics. This kind of attack is usually not easily directly identified in the time domain, and its spectrum energy distribution often has more obvious regularity. To capture this information, the frequency–domain branch transforms the TCN backbone output along the temporal axis and learns discriminative patterns over frequency bins. Importantly, the branch deliberately discards the Fourier phase; therefore, its final output is treated as a learned spectral embedding rather than a reconstructed temporal signal. The overall processing flow of the frequency-domain branch is illustrated in Figure 5 and is divided into four steps:
  **(1)**  **rFFT.** A one-dimensional rFFT is performed independently for each batch element and latent channel:
    (4) $$X_{b,c,f}=\sum_{t=0}^{T-1}H_{tcn,b,c,t}\cdot e^{-\frac{j2\pi ft}{T}},\;\;X\in C^{B\times C\times F}$$
    where b∈{1,2,…,B}, c∈{1,2,…,C}, and f∈{0,1,…,F−1}, with C=256 and $F=floor\frac{T}{2}+1$. Here, t indexes temporal positions and f indexes the retained non-negative frequency bins. Because the input is real-valued, the omitted negative-frequency coefficients are determined by Hermitian symmetry. The upper summation limit is T−1; F specifies the number of stored spectral coefficients.
  **(2)**  **Magnitude Spectrum Extraction.** The regular characteristics of periodic attack patterns are primarily manifested in the energy distribution of frequencies, with the spectrum exhibiting discrete peaks rather than phase information. The amplitude spectrum is obtained by discarding the Fourier phase and retaining the amplitude of each frequency component.
    (5) $$A_{b,c,f}=\left|X_{b,c,f}\right|=\sqrt{{\left(ReX_{b,c,f}\right)}^{2}+{\left(ImX_{b,c,f}\right)}^{2}}$$
    where $A\in R_{+}^{B\times C\times F}$. This representation preserves spectral amplitude information while discarding phase-dependent temporal localization.
  **(3)**  **Spectral Feature Modeling over Frequency Bins.** Two depthwise-separable residual convolution blocks are applied along the ordered frequency bin axis to model local spectral patterns and interactions among neighboring frequency components. Although Conv1D is used, these blocks are not temporal TCN blocks because their last dimension represents frequency rather than time. Denoting the learnable spectral transformation by $S_{\theta }(\cdot )$, the output is $\hat{A}=S_{\theta }(A)\in R^{B\times C\times F}$. After frequency domain modeling, a more discriminative representation of frequency spatial features can be obtained, thereby helping the model better notice repeated attack patterns.
  **(4)**  **Inverse Refactoring.** Because the original phase has been discarded, a one-sided spectrum with zero imaginary components is therefore constructed and passed to irFFT with the output length explicitly fixed to n = T:
    (6) $$\hat{A}=S_{\theta }\left(A\right)\;\to \;\tilde{X}=\hat{A}+j0\;\to \;H_{freq}=irFFT\left(\tilde{X},n=T\right)\in R^{B\times C\times T}$$

This operation is used to map the learned spectral coefficients to a real length-T embedding that can be fused with $H_{time}$. Setting the imaginary component to zero does not restore the discarded phase; if a learned real coefficient is negative, its corresponding complex phase is π rather than 0.

**3.**  **Complementarity of Dual Branches.** The dual-branch structure models the same input signal from different perspectives. The time–domain branch looks at short-term changes over time and patterns that continue for a long time, effectively capturing time–domain pulse-type attacks characterized primarily by sudden changes. Conversely, the frequency–domain branch emphasizes the analysis of frequency energy distribution and periodic variation patterns, making it more suitable for identifying attack patterns with periodic characteristics. Accordingly, the dual-branch design provides a mechanism for complementary representation learning rather than a guarantee that the frequency branch is beneficial for every dataset. The subsequent gate can adaptively combine the two shape-compatible representations, enabling dynamic collaboration across domains and enhancing the integral detection performance of the model for complex industrial network attacks.

### 3.4. Gated Time–Frequency Fusion

**Figure 6:**
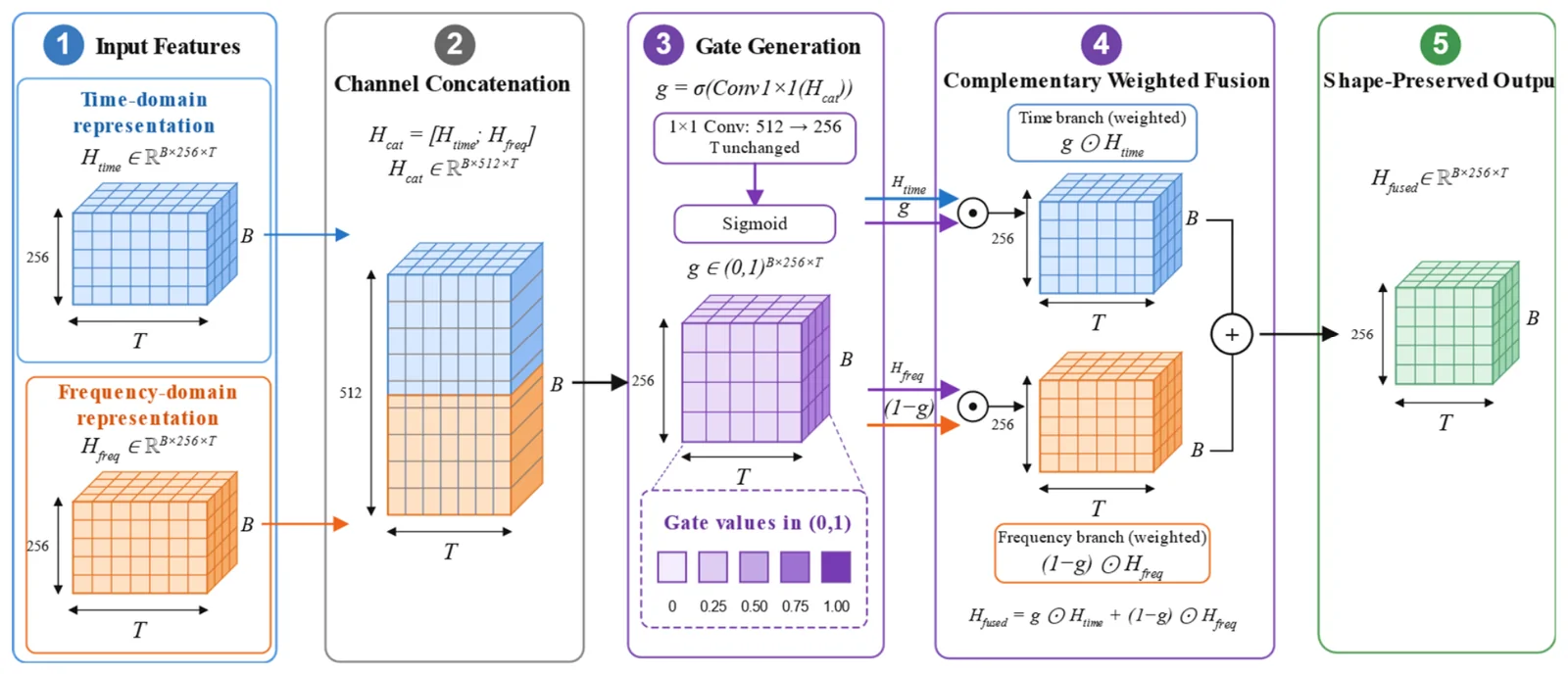
Gated fusion mechanism of time–domain and frequency–domain features. Blue and orange denote the time-domain and frequency-domain representations, purple denotes gate generation and weighted fusion, green denotes the fused output, and ⊙ and + indicate element-wise multiplication and addition, respectively.

Different attack modes exhibit varying degrees of dependence on the information of time–domain and frequency–domain. If fixed weights or direct feature concatenation are used for fusion, it is often difficult to fully leverage the synergistic advantages of the two types of features. Therefore, TFPAG-Net uses a learnable gated fusion layer to combine the time–domain and frequency–domain representations. Let $H_{time},\;H_{freq}\in R^{B\times 256\times T}$. The gate is generated from their joint latent representation and assigns a mixing coefficient to each batch element, latent channel, and length coordinate. The gated fusion mechanism is illustrated in Figure 6.

**1.**  **Gated Fusion.** The core idea of gated fusion is to dynamically allocate the commitment proportion of time–domain features and frequency–domain features across different latent channels and aligned length coordinates. By learning gated vectors, each element represents the relative contribution of the time–domain and frequency–domain representations at the corresponding latent channel and aligned length coordinate. The particular calculation is as follows:

The time–domain and frequency–domain features are first concatenated along the channel axis:

(7) $$H_{cat}={Concat}_{channel}\left(H_{time},H_{freq}\right)\in R^{B\times 512\times T}$$

A 1×1 convolution maps the 512-channel joint representation to 256 channels, and a sigmoid function bounds the gate:

(8) $$g=\sigma \left[{Conv}_{1\times 1}\left(H_{cat}\right)\right]\in {\left(0,1\right)}^{B\times 256\times T}$$

where σ(·) is the sigmoid activation function. When the gating value approaches 1, the model pays more attention to time–domain information; when the gating value approaches 0, it relies more on frequency–domain features. This interpretation applies to learned latent coordinates and should not be regarded as a direct physical attribution to a particular sensor or frequency component. The time–domain features are multiplied by the gated weights, and the frequency–domain features are multiplied through the complementary weights and then added together, where ⊙ represents element-wise multiplication:

(9) $$H_{fused}=g⊙H_{time}+\left(1-g\right)⊙H_{freq}\in R^{B\times 256\times T}$$

**2.**  **Advantages of Gated Fusion.** Compared with a fixed mixing coefficient, the gated fusion suggested in this paper offers these benefits:
  **(1)**  **Adaptive Weight Learning.** The gating vector is automatically learned by the network, enabling its weights to dynamically adjust according to input samples. This eliminates the need for manually setting the fusion ratio between time–domain and frequency–domain features, allowing the model to automatically assign contributions of the two feature types based on their importance for different attack patterns.
  **(2)**  **Shape-Preserving Parameterization.** The 1×1 projection generates 256 gate channels without changing the length T, so the fused representation remains compatible with the following multi-scale module while introducing only a limited additional parameter cost.
  **(3)**  **Differentiable Cross-Branch Fusion.** Element-wise weighting maintains differentiable paths through both branches and permits end-to-end optimization. These architectural properties explain how the representations are combined, but they do not by themselves establish a universal performance advantage for the dual-branch design.

### 3.5. Multi-Scale Temporal Pyramid

The multi-scale temporal pyramid receives the gated feature $H_{fused}\in R^{B\times 256\times 128}$ and summarizes it at three temporal resolutions without flattening the channel and temporal dimensions. The present implementation is defined for the experimental window length T=128 and uses fixed, non-overlapping pooling factors; therefore, it is not described as window-length-independent adaptive pooling. Pooling acts only on the last dimension, while the batch and channel dimensions are preserved.

**1.**  **Multi-Scale Temporal Pooling.** Three resolutions are constructed from the same fused tensor. Because the kernel size equals the stride at each scale, the pooled regions do not overlap in the stated configuration. The scale labels refer to the relative temporal resolution of the retained bins.
  **(1)**  **Coarse Scale Capture.** Max pooling with kernel size and stride 8 summarizes each eight-position region by its strongest activation and produces 16 temporal bins:
    (10) $$P_{coarse}=\mathrm{MaxPool}\left(H_{fused};k=8,s=8\right)\in R^{B\times 256\times 16}$$
  **(2)**  **Intermediate Scale Capture.** Average pooling with kernel size and stride 4 summarizes each four-position region and produces 32 temporal bins:
    (11) $$P_{mid}=\mathrm{AvgPool}\left(H_{fused};k=4,s=4\right)\in R^{B\times 256\times 32}$$
  **(3)**  **Fine Scale Capture.** Average pooling with kernel size and stride 2 retains a denser summary of the fused sequence and produces 64 temporal bins:
    (12) $$P_{fine}=\mathrm{AvgPool}\left(H_{fused};k=2,s=2\right)\in R^{B\times 256\times 64}$$
**2.**  **Shared Channel Projection and Cross-Scale Concatenation.** Let φ(·) denote the shared 1×1 channel projection from 256 to $C_{p}=128$ channels. Applying the same projection to the fine, intermediate, and coarse tensors gives $\varphi (P_{fine})\in R^{B\times 128\times 64}$, $\varphi (P_{mid})\in R^{B\times 128\times 32}$, and $\varphi (P_{coarse})\in R^{B\times 128\times 16}$. These tensors are concatenated along the temporal bin axis only; no flattening or channel-wise concatenation is performed:
  (13) $$H_{pyr}={Concat}_{bin}\left[\varphi \left(P_{fine}\right),\varphi \left(P_{mid}\right),\varphi \left(P_{coarse}\right)\right]\in R^{B\times 128\times 112}$$
**3.**  **Pyramid Descriptor and Attention Interface.** Equation (13) yields 64+32+16=112 temporal bins while keeping the latent channel dimension fixed at 128. A sample-level descriptor is then obtained through global average pooling over the 112-bin axis:
  (14) $$h_{pool}=\frac{1}{112}\sum_{l=1}^{112}H_{pyr,b,c,l}\in R^{B\times 128}$$

The vector $h_{pool}\in R^{B\times 128}$ is used only to generate the sample-dependent channel attention logits in Section 3.6; it is not the final classification descriptor. The attention weights are broadcast over the 112 bins and reweight $H_{pyr}$ to produce $H_{out}\in R^{B\times 128\times 112}$. The classifier subsequently uses $h_{cls}=GAP(H_{out})$. Because the same channel weight is applied to every bin, the interface between the pyramid, the attention module, and the classifier is mathematically explicit. In Equation (14), every retained bin receives equal weight; consequently, the fine, intermediate, and coarse scales contribute in proportion to 64:32:16 rather than being implicitly treated as equally weighted scales.

### 3.6. Auxiliary PCMCI-Derived Association Guidance

**Figure 7:**
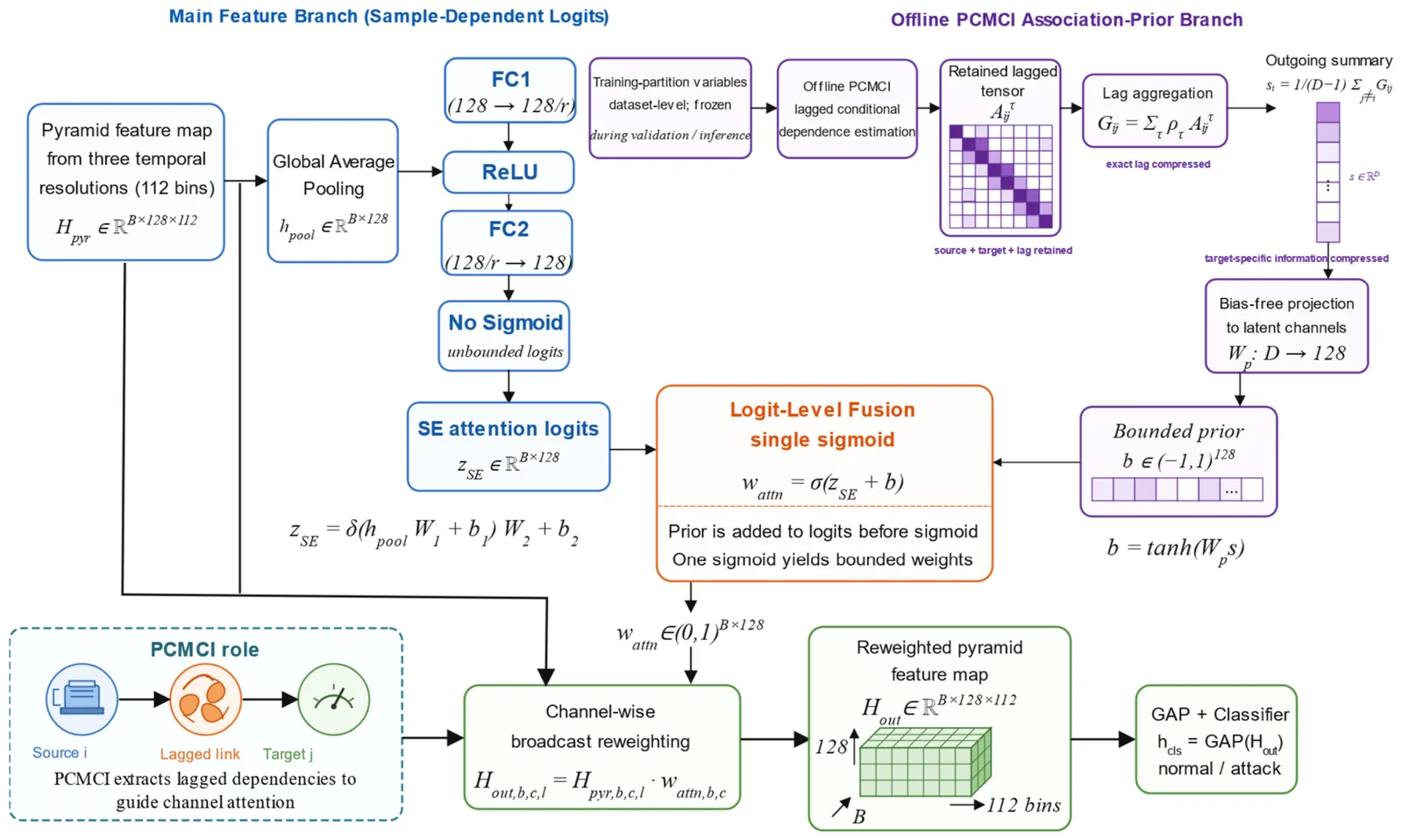
PCMCI-based association-guided attention module and information flow. Blue denotes the sample-dependent attention branch, purple denotes the offline PCMCI-derived association-prior branch, orange denotes logit-level fusion, and green denotes feature reweighting and classification; arrows indicate information flow.

The channel attention mechanism combines sample-dependent neural attention with an auxiliary training-derived association prior. In the present implementation, PCMCI is selected to estimate this prior because its conditional-dependence testing can account for multivariate context and lagged relationships that are not explicitly represented by simple pairwise correlation measures. Previous comparative studies have shown that PCMCI or its extension PCMCI+ can reduce spurious associations relative to classical correlation-based analysis in multivariate time-series settings [40,41]. Based on this methodological motivation, PCMCI is used here as an offline estimator of lagged conditional dependence rather than as a principal architectural contribution or as a method that guarantees physical causality. The overall structure of the PCMCI-based association-guided attention module is illustrated in Figure 7.

**(1)**  **Directed Lagged Association Representation.** Let D denote the number of original input variables and let the candidate lag set be $T_{lag}=\{1,\ldots ,\tau_{max}\}$. For an ordered source–target pair (i, j) and lag τ, $v_{ij}^{\tau }$ denotes the PCMCI conditional-dependence statistic between $X_{t-\tau }^{i}$ and $X_{t}^{j}$ after conditioning on the selected context and $p_{ij}^{\tau }$ denotes the significance value used for the corresponding conditional-independence decision. The retained association magnitude is
  (15) $$A_{ij}^{\tau }=\left|v_{ij}^{\tau }\right|\;I\;\left[p_{ij}^{\tau }<\alpha \right],\;\;\tau \in T$$
  where α is the significance threshold and I [·] is the indicator function. Taking the absolute value intentionally discards the sign of the conditional-dependence statistic because the prior encodes association importance rather than effect sign. At this stage, source–target ordering is retained by the ordered pair (i, j), and lag information is retained by τ.

The retained lagged associations are first aggregated into a directed pairwise matrix:

(16) $$G_{ij}=\frac{\sum_{\tau \in T_{lag}}\rho_{\tau }A_{ij}^{\tau }}{\sum_{\tau \in T_{lag}}\rho_{\tau }},\;\;\sum_{\tau \in T}\rho_{\tau }\geq 0$$

where $\rho_{\tau }\geq 0$ denotes the weight assigned to lag τ. In this study, no prior preference is imposed on different candidate lags; therefore, equal lag weights are adopted, i.e., $\rho_{\tau }=1$ for all τ∈T. Consequently, Equation (16) is equivalent to averaging the retained conditional-dependence magnitudes over the candidate lag set. The outgoing association strength of source variable i is then computed as

(17) $$s_{i}=\frac{1}{D-1}\sum_{j\neq i}G_{ij}$$

These stages retain different amounts of structural information. $A_{ij}^{\tau }$ retains source, target, and lag; $G_{ij}$ retains source-to-target pair orientation but compresses the exact lag; and the final outgoing vector s retains only aggregate source role information and therefore no longer identifies the specific target or lag responsible for a large value. This centrality compression is an explicit information–efficiency trade-off and should not be described as a fully lag- and direction-aware representation.

This compression is adopted to avoid introducing a $D\times D\times \tau_{max}$ structural tensor into the latent attention module, which would substantially increase the parameter and memory cost and would require additional mapping between original variables, pairwise edges, lags, and latent channels.

**(2)**  **PCMCI Configuration.** PCMCI was applied exclusively to the training partition of each dataset with a maximum lag of $\tau_{max}=5$. The conditional-independence test was implemented using ParCorr, with a significance threshold of α=0.1. For each ordered source–target pair $\left(i,j\right)$ and candidate lag τ, an association was retained only when the corresponding significance value satisfied $p_{ij}^{\left(\tau \right)}<\alpha$. Equal weights were assigned to all candidate lags when constructing the directed pairwise representation $G_{ij}$. PCMCI estimation was performed only once on the training data, and the resulting association prior was subsequently frozen during validation and test inference.
**(3)**  **Variable-to-Latent Prior Projection.** The association vector s is defined over the D original variables, whereas the pyramid representation contains $C_{p}=128$ latent channels. These spaces do not have a natural one-to-one correspondence. A learnable bias-free projection therefore maps the variable-level summary into the latent attention space. The bounded channel space prior is
  (18) $$b=tanh\left(W_{p}s\right)\in {\left(-1,1\right)}^{C_{p}},\;\;W_{p}\in R^{C_{p}\times D}$$

The projection does not imply that an original sensor variable corresponds directly to a particular latent channel. Instead, it converts the dataset-level association summary into a bounded latent context vector jointly used by the detector. Because the projection is bias-free, s=0 implies b=0; thus, the association module does not introduce an arbitrary constant offset when no retained association is present. The projected vector b should not be interpreted as a direct sensor-level causal attribution.

**(4)**  **Bounded Context-Guided Channel Attention.** Global average pooling over the 112 pyramid bins yields $h_{pool}\in R^{B\times 128}$. Two fully connected layers generate sample-dependent squeeze-and-excitation logits $z_{SE}$ [43], whereas the bounded prior b defined in Equation (18) is shared across samples from the same dataset:
  (19) $$Z_{SE}=\delta \left(h_{pool}W_{1}+b_{1}\right)W_{2}+b_{2}\in R^{B\times C_{p}}$$
  where $\delta \left(\cdot \right)$ denotes the ReLU activation function, $C_{p}=128$ is the number of pyramid channels, r is the SE reduction ratio, $W_{1}\in R^{C_{p}\times C_{p}/r}$, and $W_{2}\in R^{C_{p}/r\times C_{p}}$. The bias vectors are broadcast along the batch dimension. No sigmoid operation is applied at this stage because $Z_{SE}$ represents unbounded channel attention logits.

Rather than multiplying a second bias term with an already sigmoidal attention weight, the association prior is added at the logit level and sigmoid is applied once:

(20) $$w_{attn}=\sigma \left(z_{SE}+b\right)\in {\left(0,1\right)}^{B\times C_{p}}$$

(21) $$H_{out,b,c,l}=H_{pyr,b,c,l}\;w_{attn,b,c}$$

where $H_{out}\in R^{B\times C_{p}\times 112}$. Because tanh(x)∈(−1, 1) and sigmoid maps real-valued logits to (0,1), the final channel weights remain bounded. Equations (20) and (21) express the channel-wise broadcast explicitly at each pyramid bin. The reweighted feature map is then globally averaged to obtain the classification descriptor $h_{cls}$; equivalently, because the same channel weight is applied to all 112 bins, $h_{cls}=h_{pool}⊙w_{attn}$. The classifier operates on this reweighted descriptor rather than on the unmodulated $h_{pool}$.

**(5)**  **Interpretive Scope.** A PCMCI edge represents lagged conditional dependence under the selected variables, lag range, and significance rule. The final variable-level summary further compresses exact lag and target-specific information. Previous comparative studies suggest that PCMCI and related multivariate conditional-dependence methods can reduce spurious associations or false-positive dependencies relative to conventional pairwise correlation analysis under their respective experimental settings [40,41]. Accordingly, PCMCI is used only as an auxiliary association prior rather than a principal contribution of TFPAG-Net.

### 3.7. Loss Function

Addressing the class imbalance problem commonly encountered in IIoT intrusion detection, this paper adopts weighted focal loss [44,45] as the training objective. To avoid notation conflict with the PCMCI significance threshold alpha, the class balancing coefficient is denoted by $\omega_{c}$ in this section.

**(1)**  **Weighted Focal Loss.** Focal loss represents an enhancement derived from the standard cross-entropy loss. By incorporating an adjustable focusing parameter γ, it diminishes the weight assigned to easily classifiable samples, thereby directing the model’s attention towards hard-to-classify samples. For N training samples, the weighted focal loss is defined as
  (22) $$L_{focal}=-\frac{1}{N}\sum_{i=1}^{N}\omega_{y_{i}}{\left(1-p_{i,y_{i}}\right)}^{\gamma }log\left(p_{i,y_{i}}\right)$$
  where $p_{i,y_{i}}$ denotes the predicted probability assigned to the true class $y_{i}$, γ≥0 is the focusing parameter, and $\omega_{y_{i}}>0$ denotes the class balancing weight associated with the true class. When γ is 0, the focal loss simplifies to the regular cross-entropy loss. When γ>0, for easy-to-classify samples ($p_{y_{i}}\to 1$), the modulation factor $(1-p_{y_{i}}{)}^{\gamma }$ approaches 0, significantly reducing the loss contribution; for difficult-to-classify samples ($p_{y_{i}}\to 0$), the modulation factor $(1-p_{y_{i}}{)}^{\gamma }$ approaches 1, retaining the loss contribution.
**(2)**  **Effective Number Class Weight.** To further alleviate class imbalance, the class balancing coefficient is determined according to the effective number of samples formulation proposed by Cui et al. [46]. For class c with $N_{c}$ training samples, its effective number is $E_{c}=\frac{\left(1-\beta^{N_{c}}\right)}{\left(1-\beta \right)}$, and the corresponding inverse effective number weight is defined as
  (23) $${\tilde{\omega }}_{c}=\frac{1}{E_{c}}=\frac{1-\beta }{1-\beta^{N_{c}}}$$

Here, β controls the saturation rate of the effective number as the class sample size increases. The resulting class weights are normalized across classes before being substituted into the weighted focal loss in Equation (22). Only the numerical class weights varied according to the class distribution of the training partition, while the weighting formulation and all associated hyperparameters remained unchanged. The resulting class weights were fixed during validation and testing.

## 4. Experiments

This section evaluates TFPAG-Net and all baseline models under explicitly documented data partition, training, and statistical protocols. Macro-F1 is treated as the primary metric because all three datasets are class-imbalanced, while accuracy is reported as a secondary metric. Unless otherwise stated, each model–dataset combination was independently trained and evaluated five times using the predefined random seeds 42, 123, 456, 789, and 1024. The train/validation/test partitions were kept fixed across the five runs, and model-level results are reported as the mean and sample standard deviation of the corresponding five test scores. We distinguish descriptive point estimates from statistically supported differences and interpret all conclusions within the limitations of the public data order, minority class support, and local baseline implementations.

### 4.1. Dataset Description

This paper evaluates TFPAG-Net on three publicly available datasets: Edge-IIoTset [47] as the primary dataset, X-IIoTID [48] as a low-dimensional complementary dataset, and SWaT [49] as an industrial control system dataset. These datasets cover different feature dimensions and application scenarios and therefore support a comparative evaluation of TFPAG-Net under the explicitly documented protocols summarized in Table 1.

**Figure 8:**
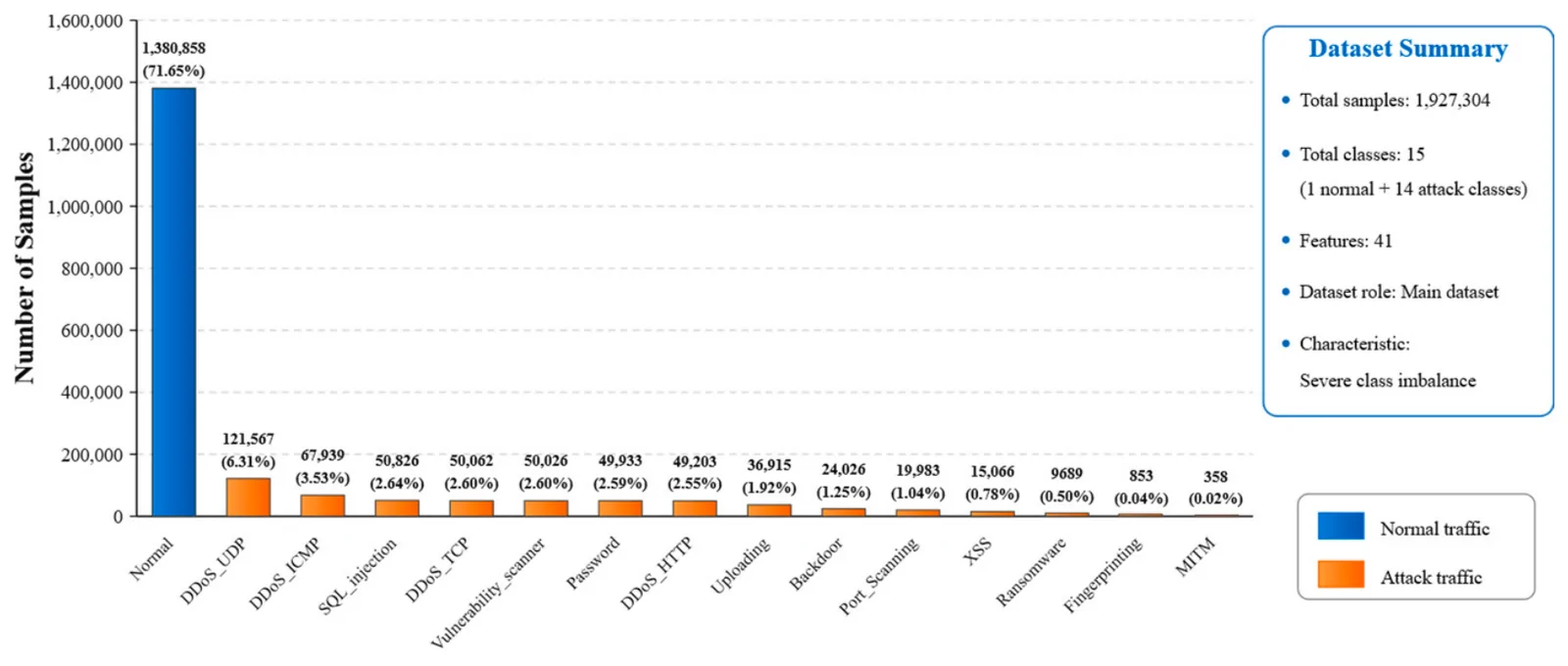
Edge-IIoTset Category Distribution.

**Figure 9:**
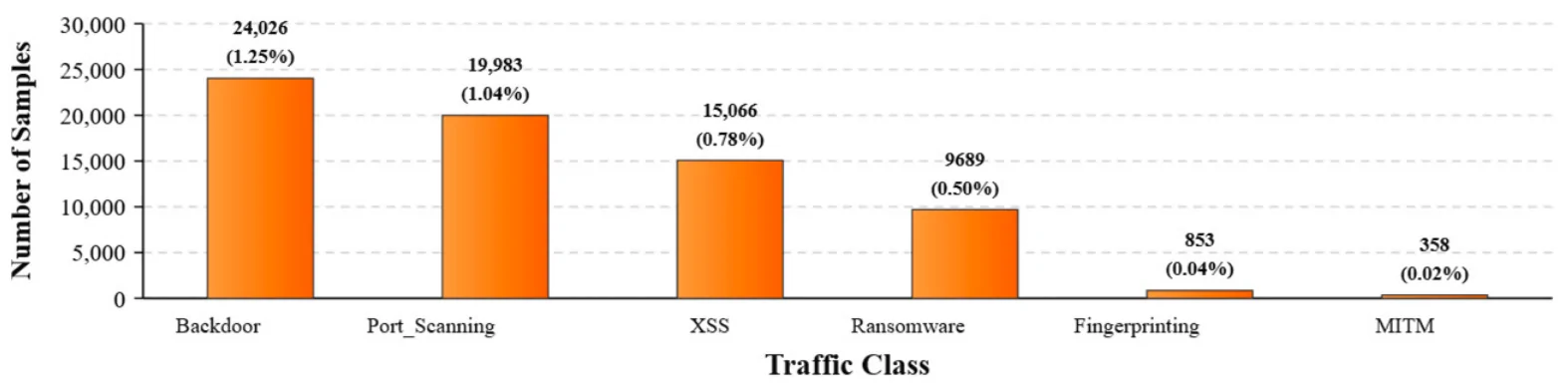
Minor details of Edge-IIoTset.

**Figure 10:**
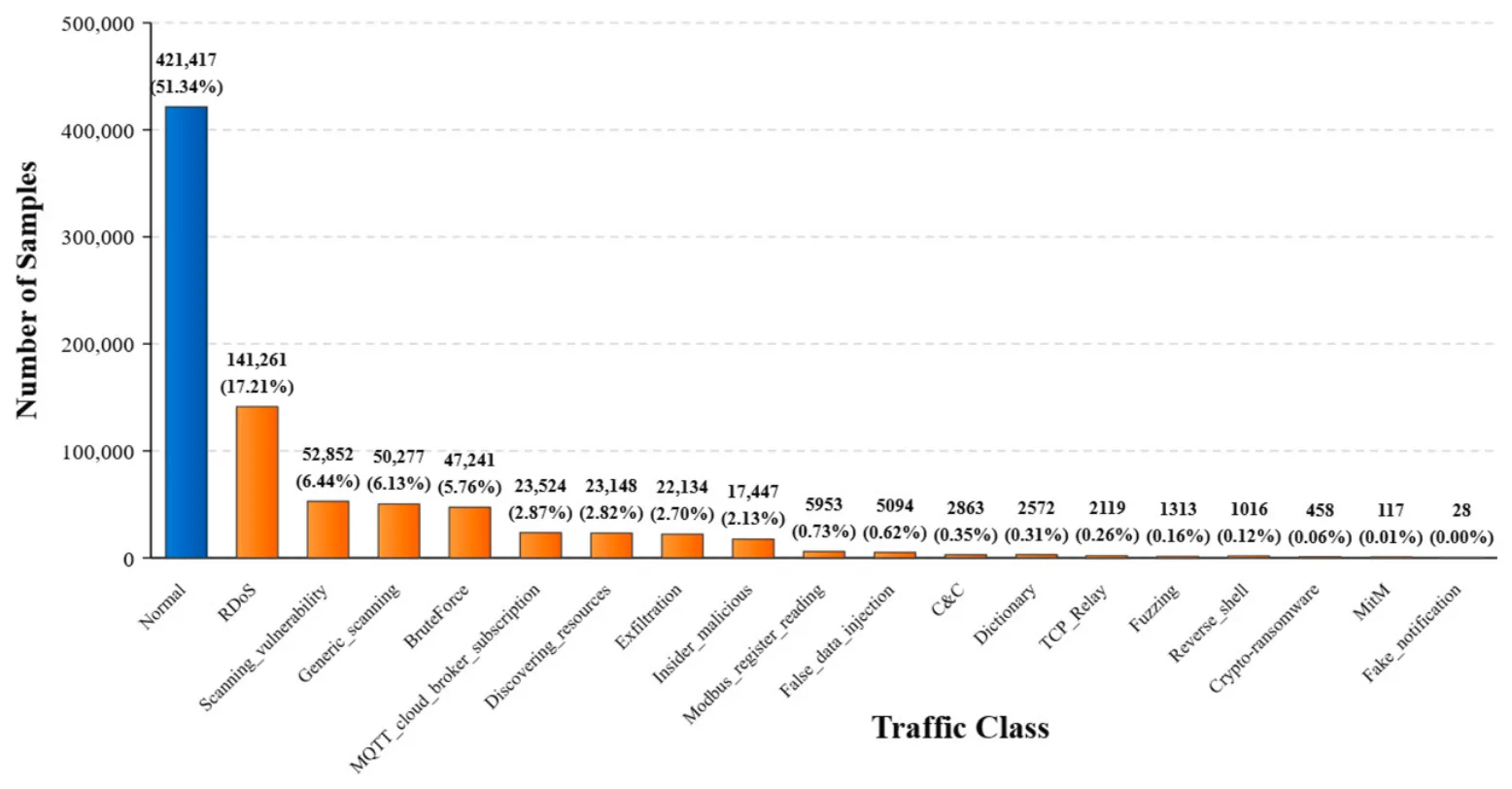
X-IIoTID category distribution. Blue denotes normal traffic, whereas orange denotes attack traffic.

**Figure 11:**
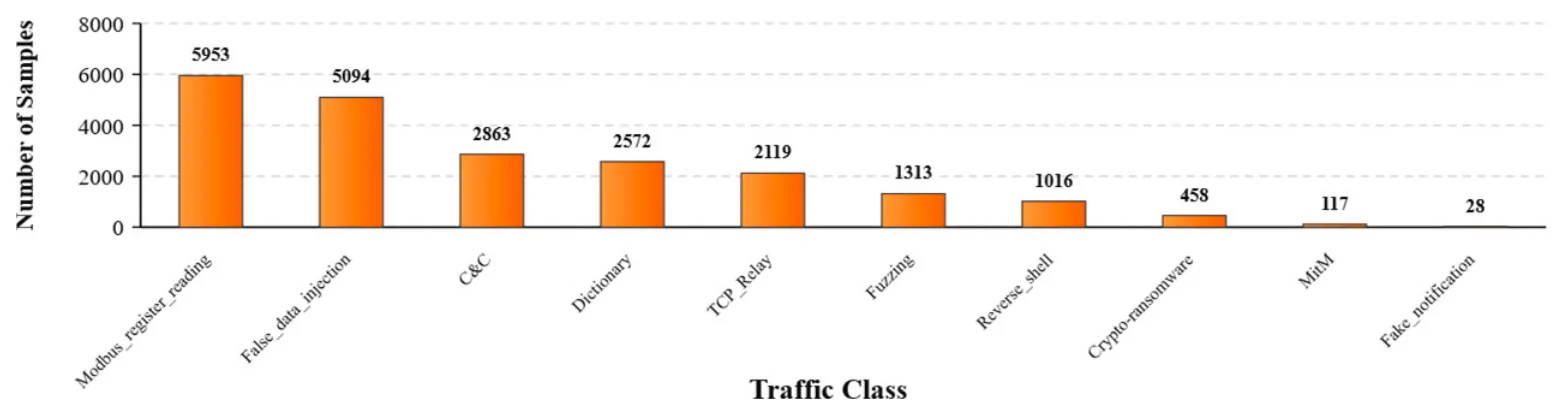
Minor details of X-IIoTID.

The specific characteristics of each dataset are as follows:

**(1)**  **Edge-IIoTset.** Developed by Ferrag et al. in 2022 [47], this dataset is specifically designed for IIoT security research purposes. It incorporates 41-dimensional features extracted from IIoT sensor networks, including sensor readings for temperature, pressure, and flow, as well as gateway communication logs. The dataset encompasses 15 categories, with attack types spanning Backdoor, various DDoS variants, Man-in-the-Middle attacks, Fingerprinting, Password attacks, PortScans, Ransomware, and SQL Injection, among others. This dataset constitutes the primary dataset utilized in this paper, enabling comprehensive baseline comparisons and ablation analyses. Rows were first assigned to mutually exclusive stratified training, validation, and test partitions (79%/11%/10%), after which class-homogeneous length-128 windows with stride 64 were formed within each partition. The released DNN table does not provide a validated continuous capture order; therefore, this protocol prevents raw row sharing across subsets but should not be interpreted as strict future time generalization. Detailed class distributions are shown in Figure 8 and Figure 9.
**(2)**  **X-IIoTID.** Introduced by Al-Hawawreh et al. in 2022 [48], X-IIoTID represents a cross-architecture dataset for IIoT intrusion detection. This dataset encompasses 18-dimensional features and 19 label categories (normal conditions plus 18 types of attacks). Its lower feature dimensionality provides a complementary setting for evaluating classification under sparse feature representations. Because the released rows are shuffled, we used the same split-before-window protocol as for Edge-IIoTset. For rare classes whose assigned partition contained fewer than T=128 rows required to construct a complete window, samples were repeated only from the same class and the same assigned partition until one length-128 window could be formed. No samples from another class or another training/validation/test partition were introduced. Therefore, this rare class handling did not create cross-partition information leakage. Several test classes consequently contain only one to three windows, and their class-wise estimates should be interpreted cautiously. The category distribution is shown in Figure 10 and Figure 11.
**(3)**  **SWaT.** Released by Goh et al. [49] from the Singapore Institute of Technology in 2017, SWaT contains 51 sensor/actuator variables and two label categories, with attacks accounting for approximately 7% of the released records. Known engineering relations among several variables allow a limited physical consistency check of lagged associations. However, SWaT does not provide ground-truth causal graphs and is not used as proof of causal discovery.

### 4.2. Data Preprocessing

To guarantee a fair comparison among all methods, this paper implements a standardized data preprocessing protocol across all datasets:

**(1)**  **Missing Value Handling.** Selected feature columns were converted to numerical values, infinite values were treated as missing, and remaining missing values were filled through linear interpolation followed by forward and backward filling.
**(2)**  **Feature Standardization.** Perform Z-score normalization independently for each feature dimension:
  (24) $${\hat{x}}_{i,t,d}=\frac{x_{i,t,d}-\mu_{d}}{\sigma_{d}}$$
  where $\mu_{d}$ and $\sigma_{d}$ denote the mean and standard deviation of feature d computed exclusively from training-owned rows. The same statistics were then applied unchanged to validation and test windows.
**(3)**  **Time Series Window Construction.** For Edge-IIoTset and X-IIoTID, rows were partitioned before windowing, and length-128 windows with stride 64 were created only within the owning partition. For SWaT, length-128 non-overlapping windows (stride 128) were formed separately within each source file and then assigned to the three subsets. Protocol audits found zero cross-subset raw row references. Of 927,149 SWaT rows, 926,976 were assigned exactly once, and 173 incomplete source-tail rows were excluded.
**(4)**  **Loss and Class Weight Configuration.** Class weights were calculated exclusively from the training labels using the effective number formulation described in Section 3.7 and normalized before being incorporated into the weighted focal loss. The focusing parameter and effective number parameter were fixed at γ=2 and β=0.9999, respectively. Although the resulting numerical class weights differ because the class distributions of the datasets are different, the weighting formulation and all associated hyperparameters remain unchanged. The computed class weights were fixed during validation and testing.

### 4.3. Baseline Method

To comprehensively evaluate TFPAG-Net, six representative baseline methods were trained using the same fixed train/validation/test targets and the same five predefined random seeds of 42, 123, 456, 789, and 1024. Architectural hyperparameters were kept model-specific but fixed across datasets. The six baseline models and their implementation details are summarized in Table 2.

Special implementation details of the baseline method:

- LSTM: Two-layer LSTM with a hidden dimension of 128, followed by a fully connected classification head.
- CNN-BiLSTM: The first layer uses one-dimensional convolution to extract local features, with 64 filters and a kernel size of 3. It is followed by two layers of bidirectional LSTM, with a hidden dimension of 128.
- Transformer-IDS: Four self-attention heads, an embedding dimension of 128, and a two-layer Transformer encoder were used.
- TimesNet: A local simplified TimesNet-style implementation was trained for at most 50 epochs with an Inception hidden dimension of 128.
- FFT-RDNet: A local simplified FFT-RDNet-style implementation used three depthwise separable convolution layers.
- GAT-IDS: The hidden dimension of the attention layer is set to 64, and the number of heads is set to 4.

The training parameters for the TFPAG-Net model and six baseline models proposed in this paper are shown in Table 3.

All models were evaluated under a unified dataset-specific training protocol. TFPAG-Net and the six baseline models used identical train/validation/test partitions, the same weighted focal loss formulation, the same class weighting strategy, the same batch size, the maximum training budget, an early stopping criterion, and a model selection rule. For Edge-IIoTset and X-IIoTID, predictions were obtained directly using the class with the highest output probability. For SWaT, a binary decision threshold was determined exclusively from the validation partition and then fixed for test evaluation. This threshold selection procedure is an evaluation rule and does not alter the unified training configuration.

### 4.4. Ablation Experiment

All ablation variants were evaluated using the same fixed data partitions, unified optimization settings, and five predefined random seeds as the full model. For Edge-IIoTset and X-IIoTID, six stored variants were compared: (1) TCN only, (2) dual branch, (3) dual branch plus pyramid, (4) TFPAG-Net Full, (5) time branch only, and (6) frequency branch only. For SWaT, the component ablation additionally includes (1) TCN + Time, (2) TCN + Frequency, (3) Pyramid + SE, and (4) Pyramid + PCMCI variants. The full-model results were obtained under exactly the same protocol as the main comparison experiments.

The following ablation analysis therefore focuses on the contribution of the integrated model components, while PCMCI is interpreted only as an auxiliary association prior rather than as an independently established source of performance improvement.

### 4.5. Model Evaluation Metrics

This article adopts the following evaluation indicators:

**(1)**  **Precision.** Precision is the number of correct positive results out of all the results the model said were positive:
  (25) $${\mathrm{Precision}}_{c}=\frac{TP_{c}}{TP_{c}+FP_{c}}$$
  where $TP_{c}$ represents the number of true positives for class c and $FP_{c}$ represents the number of false positives.
**(2)**  **Recall.** Recall rate is the proportion of precisely identified positive samples of class c by the model:
  (26) $${\mathrm{Recall}}_{c}=\frac{TP_{c}}{TP_{c}+FN_{c}}$$
  where $FN_{c}$ represents the number of false negatives in category c.
**(3)**  **F1 Score.** The F1 score represents the harmonic average of recall and precision, serving as a comprehensive metric for evaluating the accuracy and completeness of a model:
  (27) $${F1}_{c}=\frac{2\times {\mathrm{Precision}}_{c}\times {\mathrm{Recall}}_{c}}{{\mathrm{Precision}}_{c}+{\mathrm{Recall}}_{c}}$$
**(4)**  **Macro F1.** The Macro-F1 metric is determined by calculating the arithmetic average of the F1 scores for each category, giving equal weight to both minority and majority classes, and is thus well-suited to evaluating the overall performance on imbalanced datasets:
  (28) $$\mathrm{Macro}\;F1=\frac{1}{C}\sum_{c=1}^{C}{F1}_{c}$$
**(5)**  **Accuracy.** Accuracy means how many times the model correctly classified the samples compared to the total number of samples:
  (29) $$\mathrm{Accuracy}=\frac{1}{N}\sum_{n=1}^{N}I(y_{n}=\hat{y_{n}})=\frac{\sum_{c=1}^{C}TP_{c}}{N}$$

### 4.6. Experimental Results and Analysis

#### 4.6.1. Main Experimental Results

This section compares TFPAG-Net with six baseline models on Edge-IIoTset, X-IIoTID, and SWaT under the unified protocol described in Section 4.3. All seven models used the same fixed data partitions, weighted focal loss, Adam optimizer with a learning rate of $1\times 10^{-3}$, batch size of 128, maximum training budget of 50 epochs, early-stopping rule, and five predefined random seeds. The test partition was evaluated only after validation-based checkpoint selection. Table 4 reports the mean ± sample standard deviation of Macro-F1 and accuracy over the five runs.

**Figure 12:**
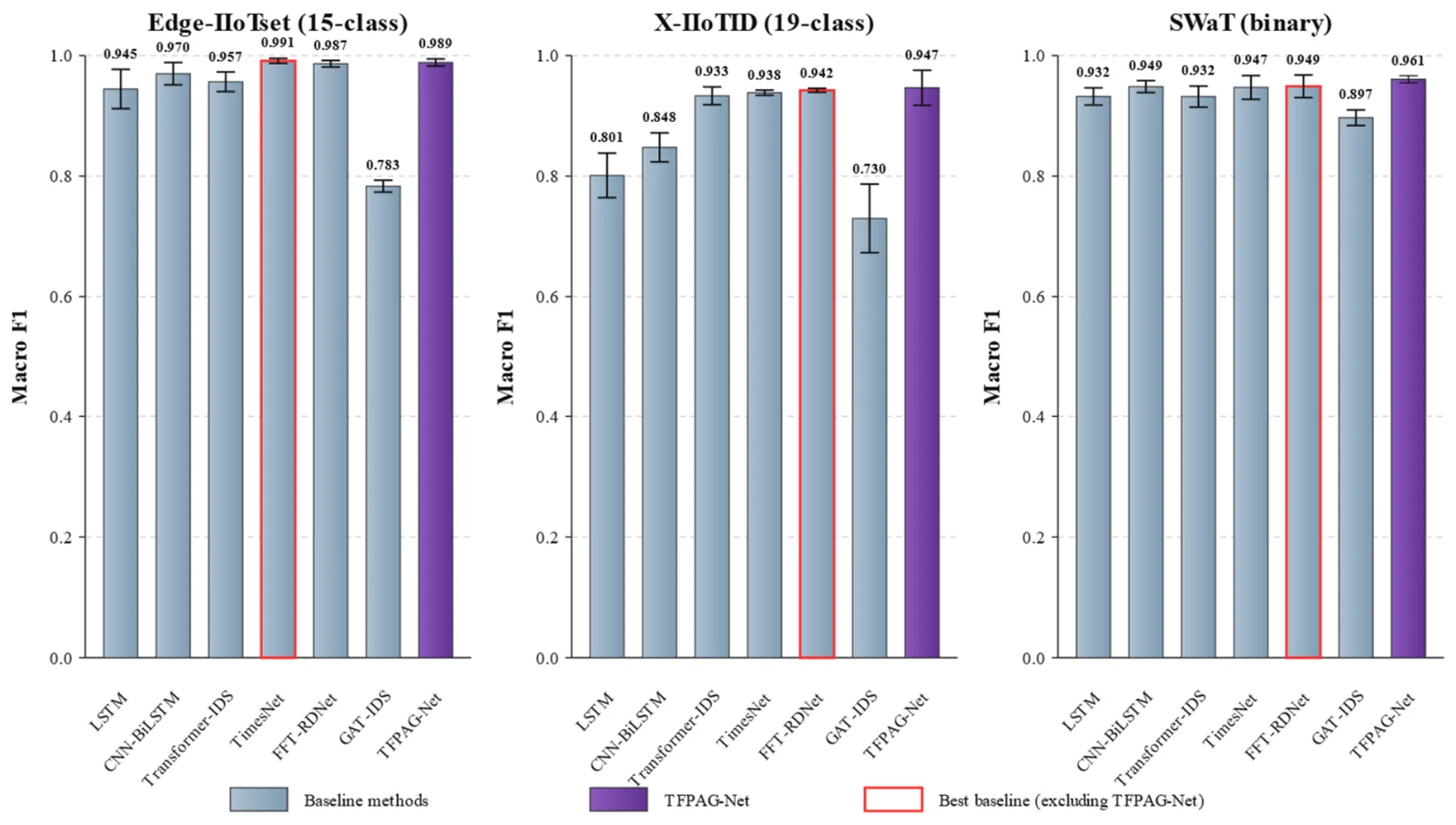
Mean Macro-F1 comparison of TFPAG-Net and six baseline models under the unified training protocol.

The main experimental comparisons across the three datasets are shown in Figure 12.

**(1)**  **Multi-classification scenario (Edge-IIoTset, 15 classes).** Under the unified five-seed training protocol, TFPAG-Net achieved a mean Macro-F1 of 0.9886 ± 0.0058 on Edge-IIoTset, ranking second among the evaluated methods. TimesNet obtained the highest mean Macro-F1 of 0.9912 ± 0.0042, corresponding to a difference of only 0.0026. The small performance gap indicates that TFPAG-Net remains highly competitive in this 15-class intrusion detection setting. The strong performance of TimesNet suggests that multi-period temporal structures provide useful discriminative information for Edge-IIoTset, whereas TFPAG-Net achieves comparable performance by jointly exploiting temporal dynamics, spectral characteristics, multi-scale information, and association-guided feature refinement. Because all models were evaluated under the same loss and optimization settings defined in Table 3, the comparison is conducted under matched training conditions. These results indicate that TFPAG-Net achieves competitive performance in the present multi-class intrusion detection setting.
**(2)**  **Multi-classification scenario (X-IIoTID, 19 classes).** Under the unified five-seed training protocol, TFPAG-Net achieved the highest mean Macro-F1 of 0.9466 ± 0.0290 among the evaluated models. The strongest baseline, FFT-RDNet, achieved 0.9423 ± 0.0033, corresponding to a mean difference of 0.0043. TFPAG-Net exhibits comparatively larger between-run variability on this dataset, indicating that optimization is more sensitive to the heterogeneous 19-class classification setting. Because all models use identical loss and optimization settings, as specified in Table 3, the observed differences are evaluated under matched training conditions. The relatively strong performance of frequency-aware models suggests that spectral characteristics provide useful discriminative information in the heterogeneous 19-class setting. Within this experimental scope, TFPAG-Net achieves competitive overall recognition performance by integrating temporal and spectral representations with gated fusion, multi-scale aggregation, and channel attention refinement.
**(3)**  **Binary classification scenario (SWaT, 2 classes).** Under the unified five-seed training protocol, TFPAG-Net achieved the highest mean Macro-F1 of 0.9607 ± 0.0059 on SWaT. The strongest baseline, FFT-RDNet, achieved 0.9490 ± 0.0187, corresponding to a mean difference of 0.0117. Most temporal- and frequency-aware models achieved relatively high performance on this binary task, indicating that SWaT presents a different classification regime from the two multi-class datasets. Under the same loss, optimization, and random seed settings defined in Table 3, TFPAG-Net maintains a competitive combination of mean performance and between-run stability. These results support the usefulness of jointly modeling temporal variation, spectral structure, multi-scale information, and association-guided refinement in the present SWaT setting without implying universal superiority across datasets.

We further compare the parameter counts and single-sample inference latency of the evaluated models to provide a same-platform computational reference. All latency measurements were obtained on an NVIDIA RTX 4070Ti with a batch size of B=1. These measurements characterize the computational behavior of the evaluated models only under the reported desktop GPU environment. The complexity of the model is shown in Table 5.

TFPAG-Net contains approximately 0.29 M parameters, which is higher than TimesNet and FFT-RDNet but lower than LSTM, GAT-IDS, Transformer-IDS, and CNN-BiLSTM. Thus, the proposed time–frequency dual-branch, multi-scale, and attention mechanisms introduce additional parameters compared with the two smallest baselines, while the overall model size remains moderate among the evaluated methods. Under the same hardware environment, TFPAG-Net achieves an inference latency of 1.6 ms compared with 1.5 ms for TimesNet and 1.1 ms for FFT-RDNet. Considering the detection results in Table 4, the additional model capacity is accompanied by improved Macro-F1 over FFT-RDNet by 0.0021, 0.0043, and 0.0117 on Edge-IIoTset, X-IIoTID, and SWaT, respectively. These results indicate a trade-off between additional model complexity and improved representation capability rather than pursuing the minimum parameter count alone.

#### 4.6.2. Ablation Experiment Analysis

**(1)**  **The ablation results and analysis of Edge-IIoTset**, which are presented in Table 6.

The Dual Branch alone does not improve performance on Edge-IIoTset. Compared with TCN Only, introducing the time–frequency dual-branch structure decreases the Macro-F1 from 0.9700 to 0.9634, corresponding to a reduction of 0.0066, while the standard deviation increases from 0.0105 to 0.0244. This suggests that simply introducing complementary temporal and frequency–domain representations does not necessarily lead to an immediate performance gain, and the interaction between heterogeneous features may introduce additional optimization variability when effective aggregation mechanisms are absent. After incorporating the multi-scale temporal pyramid, the Macro-F1 increases from 0.9634 to 0.9741, yielding an improvement of 0.0107 and exceeding TCN Only by 0.0041. Meanwhile, the standard deviation decreases substantially to 0.0072, indicating that multi-scale aggregation not only improves recognition performance but also stabilizes the utilization of time–frequency features. Introducing the complete channel attention module, which combines sample-dependent SE-based logits with the auxiliary PCMCI-derived association prior, further increases the Macro-F1 from 0.9741 to 0.9886, corresponding to an additional gain of 0.0145. Because separate Pyramid + SE and Pyramid + PCMCI variants are unavailable for Edge-IIoTset, this increment represents the joint effect of the complete attention module and cannot be attributed specifically to the PCMCI-derived prior. The external evidence discussed in Section 2.3 provides methodological motivation for using PCMCI as the association estimator, but the present Edge-IIoTset ablation is interpreted only as evidence for the complete attention stage rather than as an isolated validation of PCMCI. Consequently, the complete TFPAG-Net achieves the best performance among the ablation configurations, with an accuracy of 0.9981 and a Macro-F1 of 0.9886. Compared with TCN Only, the complete model improves Macro-F1 by 0.0186, indicating that the overall gain arises from the interaction among time–frequency representation learning, multi-scale aggregation, and association-guided channel refinement. Furthermore, the Time Branch Only and Freq Branch Only variants achieve Macro-F1 scores of 0.9458 and 0.9554, respectively, both considerably lower than the complete model. Notably, the frequency–domain branch performs better than the time–domain branch when used independently, indicating that spectral information also contains useful discriminative characteristics for Edge-IIoTset. However, neither single-domain representation is sufficient to achieve optimal performance, further supporting the effectiveness of jointly modeling temporal and frequency–domain characteristics and subsequently refining their interaction through multi-scale aggregation and association-guided attention.

**(2)**  **The ablation results and analysis of X-IIoTID**, which are presented in Table 7.

The dual-branch architecture provides a moderate improvement on X-IIoTID. Compared with TCN Only, introducing the time–frequency dual branches increases the Macro-F1 from 0.9227 to 0.9301, corresponding to a gain of 0.0074. This suggests that complementary temporal and frequency–domain representations can provide additional discriminative information for the 19-class intrusion detection task, although the increase in standard deviation from 0.0134 to 0.0298 indicates that directly combining the two feature domains may introduce additional optimization variability. After incorporating the multi-scale temporal pyramid, the Macro-F1 further increases from 0.9301 to 0.9395, yielding an additional improvement of 0.0094, while the standard deviation decreases substantially to 0.0046. This indicates that multi-scale aggregation is beneficial for X-IIoTID, as it not only captures temporal patterns over different receptive ranges but also improves the stability of the fused time–frequency representations. Introducing the complete channel attention module, which combines SE-based sample-dependent attention logits with the PCMCI-derived bounded association prior, further raises the Macro-F1 from 0.9395 to 0.9466, corresponding to an additional gain of 0.0071. The complete TFPAG-Net therefore achieves the highest Macro F1 among the X-IIoTID ablation configurations, with an accuracy of 0.9983, and improves Macro F1 by 0.0239 compared with TCN Only. Because separate Pyramid + SE and Pyramid + PCMCI variants were not retained for X-IIoTID, the 0.0071 improvement introduced at the final stage represents the joint effect of the complete channel attention module and cannot be attributed independently to PCMCI. Accordingly, the X-IIoTID experiment supports the integrated attention mechanism rather than PCMCI as an isolated contributor. The PCMCI-derived prior remains an auxiliary component whose selection is methodologically motivated by the comparative literature discussed in Section 2.3. Furthermore, the Freq Branch Only variant achieves a Macro F1 of 0.9375, outperforming the Time Branch Only variant at 0.9187 and also exceeding the Dual Branch configuration at 0.9301. This observation indicates that frequency–domain characteristics contain particularly useful discriminative information for X-IIoTID. Nevertheless, the frequency-only configuration still remains below the complete model, indicating that the best performance under the present X-IIoTID setting is obtained through the integrated combination of time–frequency representations, multi-scale temporal aggregation, and the complete channel attention module.

**(3)**  **The ablation results and analysis of SWaT**, which are presented in Table 8.

On the SWaT binary classification task, both temporal and frequency–domain modeling contribute positively to detection performance. Compared with TCN Only, introducing the temporal branch increases the Macro-F1 from 0.9356 to 0.9505, corresponding to an improvement of 0.0149, whereas introducing the frequency–domain branch raises it to 0.9448, with a gain of 0.0092. The temporal branch therefore provides a larger individual contribution on SWaT, suggesting that local temporal variations are particularly informative for distinguishing abnormal industrial process behaviors. Combining the two branches further increases the Macro-F1 to 0.9512, exceeding the temporal-only and frequency-only configurations by 0.0007 and 0.0064, respectively. This indicates that temporal and frequency–domain representations provide complementary information, although the additional gain over the stronger temporal branch is relatively limited. Adding the multi-scale temporal pyramid further increases the Macro-F1 from 0.9512 to 0.9519, corresponding to a modest improvement of 0.0007. The SWaT ablation further shows that the effect of association-guided refinement depends on the characteristics of the dataset and should not be interpreted as evidence that PCMCI is generally superior to simpler statistical association measures. The selection of PCMCI is instead motivated by previous studies in other multivariate time-series domains, where conditional-dependence-based methods were reported to reduce spurious associations or false-positive dependencies relative to conventional correlation-based analysis [40,41]. Because these findings are external to the present IIoT setting, PCMCI is interpreted only as an auxiliary estimator within the attention module. The principal architectural contribution therefore remains the integration of temporal and frequency–domain representation learning, gated fusion, and multi-scale temporal aggregation. Overall, the complete TFPAG-Net improves Macro-F1 by 0.0251 compared with TCN Only. Although the Dual Branch + Pyramid variant achieves slightly higher accuracy of 0.9903 than the complete model at 0.9889, the complete TFPAG-Net obtains the highest Macro-F1, indicating more balanced classification performance across classes under the class-imbalanced SWaT setting. These results demonstrate that performance improvement on SWaT arises from the combined interaction of temporal and frequency–domain representation learning, multi-scale aggregation, and association-guided channel refinement.

#### 4.6.3. Training Curve and Convergence Analysis

**Figure 13:**
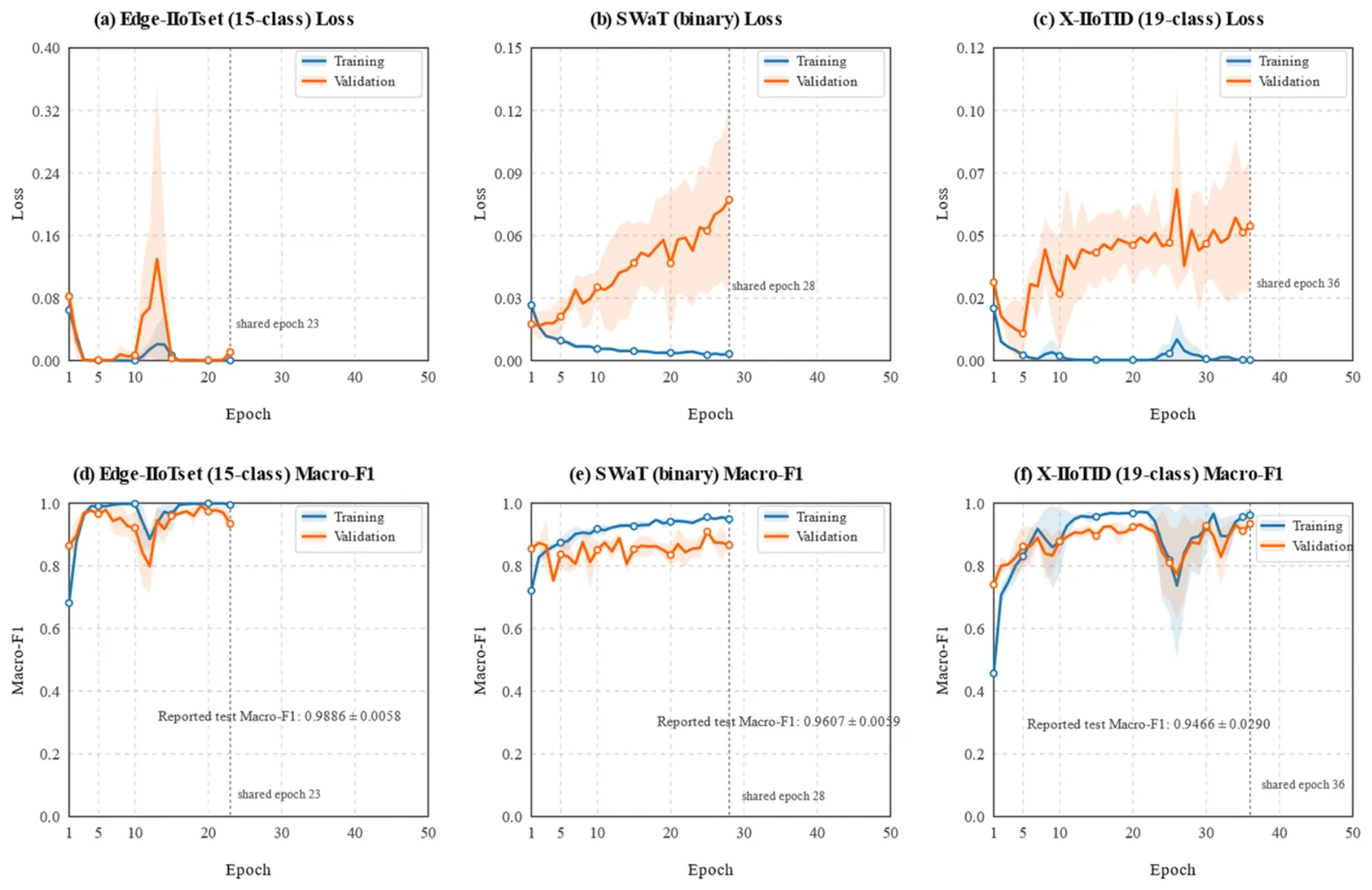
Training and validation loss and Macro-F1 curves of TFPAG-Net on the three datasets under a maximum training budget of 50 epochs.

During model training, we monitored the training and validation losses together with the corresponding Macro-F1 scores for each epoch. Figure 13 presents the loss and Macro-F1 trajectories on Edge-IIoTset, SWaT, and X-IIoTID, allowing the convergence characteristics of TFPAG-Net under different intrusion detection scenarios to be compared. Clear differences can be observed across the three datasets.

The three datasets exhibit distinct optimization characteristics. Edge-IIoTset shows the fastest and most stable convergence, with both training loss and Macro-F1 reaching favorable levels within a relatively small number of epochs. In comparison, the separation between the training and validation curves is more evident on SWaT. Although the training loss continues to decrease and the training Macro-F1 gradually increases, the validation loss rises moderately and the validation Macro-F1 fluctuates within a relatively narrow range, suggesting a certain degree of later-stage overfitting. X-IIoTID exhibits larger optimization variability, particularly around the middle stage, where a temporary increase in validation loss is accompanied by a decrease in Macro-F1 before subsequent recovery. For consistency with the main comparison, the corresponding test results are reported using the same five-seed aggregation as Table 4, yielding Macro-F1 values of 0.9886 ± 0.0058, 0.9607 ± 0.0059, and 0.9466 ± 0.0290 for Edge-IIoTset, SWaT, and X-IIoTID, respectively.

Overall, the rapid decrease in training loss and the simultaneous increase in Macro-F1 during the early epochs demonstrate that TFPAG-Net can effectively learn discriminative representations on all three datasets. However, the later-stage trajectories also show that continued reduction of the training loss does not necessarily correspond to further improvement in validation performance. This phenomenon is particularly evident on SWaT and X-IIoTID, where the validation loss exhibits larger fluctuations after the model has already reached a relatively high Macro-F1. Consequently, retaining the checkpoint corresponding to favorable validation performance is important for preventing later-stage optimization from degrading generalization. The different shared epochs of 23, 28, and 36 for Edge-IIoTset, SWaT, and X-IIoTID, respectively, further indicate that the required convergence time depends on the complexity and distribution characteristics of each dataset. These curves demonstrate that TFPAG-Net achieves effective convergence across different intrusion detection scenarios, although optimization stability becomes more challenging as the classification task becomes more complex.

#### 4.6.4. Limitations and Scope

The present study should be interpreted within the scope of the available datasets and evaluation protocols. First, the released Edge-IIoTset and X-IIoTID tables do not retain a fully validated continuous capture order. Therefore, the current within-class window construction is primarily intended to evaluate classification capability under controlled experimental conditions rather than simulating strict real-time temporal deployment. Second, some minority categories contain only a limited number of test windows, and additional samples would further strengthen the reliability of class-wise performance estimates. Third, for SWaT, source-local non-overlapping stratified windows were adopted to prevent raw row overlap between partitions. Although this protocol provides a controlled evaluation setting, future work may further examine performance under strictly chronological future period testing with a larger number of attack windows. Fourth, all main baseline comparisons were independently repeated using five predefined random seeds under the unified training protocol specified in Table 3, and the corresponding results are reported as mean ± sample standard deviation. For SWaT, the validation-derived binary decision threshold was used only as an evaluation rule and did not alter the training configuration. Therefore, the remaining limitations of the baseline comparison mainly concern the use of local model implementations and the lack of broader cross-implementation validation, rather than insufficient repeated run evaluation or inconsistencies in the training protocol. Fifth, the PCMCI-derived prior is used as an auxiliary lagged conditional-dependence prior rather than a validated causal graph or a principal methodological contribution. Its selection is motivated by previous comparative studies showing that PCMCI and related multivariate conditional-dependence approaches can reduce spurious associations or false-positive dependencies relative to conventional correlation-based analysis in other multivariate time-series domains [40,41]. Because these findings were obtained under different application settings, they are used only to motivate the estimator choice and are not interpreted as establishing universal superiority in IIoT intrusion detection. Accordingly, the main contribution of TFPAG-Net is attributed to its time–frequency dual-branch representation, gated fusion, and multi-scale temporal modeling, while the PCMCI-derived prior provides only supplementary association guidance. Finally, computational performance is hardware dependent. The parameter count and inference latency reported in Table 5 characterize the relative computational behavior of the evaluated models under the same experimental platform, whereas practical runtime characteristics may vary across different hardware architectures and software environments. Therefore, parameter count, latency, memory consumption, throughput, and computational efficiency should be considered jointly when evaluating practical implementation.

Overall, these considerations define the current experimental scope rather than detracting from the effectiveness of TFPAG-Net. The results consistently demonstrate that the proposed framework can integrate temporal, frequency–domain, multi-scale, and association-guided representations across multiple IIoT intrusion-detection datasets. At the same time, the observed dataset-dependent behavior highlights the importance of adapting individual components to different data characteristics. Future work will therefore focus on broader chronological validation, expanded minority class evaluation, cross-implementation baseline reproducibility evaluation, further analysis of association priors, and deployment-oriented testing on embedded platforms.

## 5. Conclusions and Future Work

This paper addresses the problems of heterogeneous attack patterns, complex variable relationships, and limited computational resources in IIoT intrusion detection and proposes a TFPAG-Net. The model employs depthwise separable dilated convolutions to construct a lightweight TCN backbone and integrates time–domain features, frequency–domain features, multi-scale temporal information, and variable association priors into a unified framework. The time–domain branch mainly captures local mutations and temporal dependencies, while the frequency–domain branch focuses on spectral amplitude and periodic patterns. A sample-dependent gating mechanism is used to dynamically adjust the contribution of the two branches. The multi-scale temporal pyramid further aggregates temporal information at different resolutions. As an auxiliary refinement to these principal architectural components, PCMCI is applied to the training partition to estimate lagged conditional-dependence information, which is incorporated as a bounded dataset-level bias for channel attention modulation.

Experimental results on Edge-IIoTset, X-IIoTID, and SWaT, obtained by independently evaluating TFPAG-Net and all baseline models over five predefined random seeds, demonstrate competitive and generally stable intrusion detection performance under the reported unified evaluation protocol. The ablation studies further indicate that the effects of individual components depend on dataset characteristics. In particular, the final attention-stage behavior on Edge-IIoTset and X-IIoTID reflects the joint effect of sample-dependent channel modulation and the PCMCI-derived prior and therefore should not be interpreted as isolated evidence for PCMCI. Previous studies in other multivariate time-series domains provide methodological support for selecting a conditional-dependence-based association estimator instead of relying solely on conventional pairwise correlation analysis. Accordingly, the principal contribution of TFPAG-Net lies in its lightweight time–frequency dual-branch representation, sample-dependent gated fusion, and multi-scale temporal modeling, whereas the PCMCI-derived prior is retained only as an auxiliary association refinement.

To further improve the applicability and robustness of TFPAG-Net, future research will mainly focus on the following two aspects:

1. Improve the adaptability of time–frequency modeling and experimental evaluation. Future work will explore dynamically adjusting the fusion intensity and computational allocation of the time–domain and frequency–domain branches according to different data characteristics. Sparse gating, structured pruning, and knowledge distillation can also be introduced to further reduce unnecessary computation. At the same time, more chronological evaluation protocols, larger minority-class test sets, additional baseline architectures, and independent reproducibility checks under the same unified training protocol will be considered to further improve the reliability of experimental comparison.
2. Further investigate auxiliary association modeling and computational efficiency. Future research will explore time-varying association structures, confidence-aware prior modulation, and lightweight mechanisms that retain additional directional and lag-specific information. Meanwhile, further structural optimization and complexity reduction will be investigated to improve the computational efficiency of the framework while maintaining its intrusion detection performance.

## Figures and Tables

**Table 1:** Overview of the three datasets.

Dataset	Raw Rows	Dimension	Category	Train/Val/Test Windows	Window Protocol
Edge-IIoTset	1,927,304	41	15	23,766/3292/2993	T = 128, S = 64; split rows first; within-class windows
X-IIoTID	820,834	18	19	10,107/1389/1262	T = 128, S = 64; split rows first; within-class windows
SWaT	927,149	51	2	5721/797/724	T = 128, S = 128; source-local, non-overlapping windows

**Table 2:** Detailed description of six baseline methods.

Baseline Model	Core Features	Reference
LSTM	Capture long-term temporal dependencies	Hochreiter et al., 1997 [16]
CNN-BiLSTM	Local spatial features and temporal dependencies between previous and subsequent frames	Ben Said et al., 2023 [17]
Transformer-IDS	Self-attention captures long-distance dependencies	Zhang et al., 2025 [20]
TimesNet	Convert FFT to a two-dimensional tensor and Inception module	Wu et al., 2023 [24]
FFT-RDNet	Frequency domain feature fusion and depthwise separable convolution	Xiang et al., 2025 [18]
GAT-IDS	Graph structure modeling of network entity relationships	Ahanger et al., 2025 [50]

**Table 3:** Unified training parameters of TFPAG-Net and the six baseline models.

Training Parameters	Models						
	TFPAG-Net	LSTM	CNN-BiLSTM	Transformer-IDS	TimesNet	FFT-RDNet	GAT-IDS
Epoch	50	50	50	50	50	50	50
Seeds	42, 123, 456, 789, 1024	42, 123, 456, 789, 1024	42, 123, 456, 789, 1024	42, 123, 456, 789, 1024	42, 123, 456, 789, 1024	42, 123, 456, 789, 1024	42, 123, 456, 789, 1024
Optimizer	Adam	Adam	Adam	Adam	Adam	Adam	Adam
Learning Rate	0.001	0.001	0.001	0.001	0.001	0.001	0.001
Batch size	128	128	128	128	128	128	128
Loss	Weighted focal loss	Weighted focal loss	Weighted focal loss	Weighted focal loss	Weighted focal loss	Weighted focal loss	Weighted focal loss
Focal γ	2	2	2	2	2	2	2
Effective-number β	0.9999	0.9999	0.9999	0.9999	0.9999	0.9999	0.9999
Early stopping	15	15	15	15	15	15	15

**Table 4:** Five-seed performance comparison between TFPAG-Net and the six baseline models (mean ± sample SD).

Models	Edge-IIoTset		X-IIoTID		SWaT	
	Macro F1	ACC	Macro F1	ACC	Macro F1	ACC
LSTM	0.9445 ± 0.0326	0.9922 ± 0.0034	0.8011 ± 0.0370	0.9585 ± 0.0091	0.9322 ± 0.0143	0.9848 ± 0.0041
CNN-BiLSTM	0.9699 ± 0.0185	0.9939 ± 0.0037	0.8477 ± 0.0241	0.9537 ± 0.0134	0.9485 ± 0.0099	0.9877 ± 0.0025
Transformer-IDS	0.9565 ± 0.0163	0.9947 ± 0.0006	0.9332 ± 0.0148	0.9936 ± 0.0050	0.9319 ± 0.0175	0.9848 ± 0.0044
TimesNet	0.9912 ± 0.0042	0.9981 ± 0.0004	0.9384 ± 0.0043	0.9979 ± 0.0006	0.9472 ± 0.0197	0.9878 ± 0.0052
FFT-RDNet	0.9865 ± 0.0054	0.9980 ± 0.0005	0.9423 ± 0.0033	0.9941 ± 0.0023	0.9490 ± 0.0187	0.9887 ± 0.0046
GAT-IDS	0.7832 ± 0.0098	0.9518 ± 0.0153	0.7298 ± 0.0569	0.9645 ± 0.0091	0.8969 ± 0.0129	0.9779 ± 0.0034
**TFPAG-Net**	**0.9886 ± 0.0058**	**0.9981 ± 0.0007**	**0.9466 ± 0.0290**	**0.9983 ± 0.0008**	**0.9607 ± 0.0059**	**0.9889 ± 0.0016**

**Table 5:** Model complexity and inference latency.

Model	Parameter Count (M)	Inference Latency (ms/Sample)
LSTM	0.52	1.2
CNN-BiLSTM	1.85	2.1
Transformer-IDS	1.05	3.8
TimesNet	0.09	1.5
FFT-RDNet	0.15	1.1
GAT-IDS	0.68	2.3
TFPAG-Net	0.29	1.6

**Table 6:** Ablation results of Edge-IIoTset.

Variant	Macro-F1 (Mean ± Sample SD)	Accuracy
TCN Only	0.9700 ± 0.0105	0.9969
Dual Branch	0.9634 ± 0.0244	0.9960
Dual Branch + Pyramid	0.9741 ± 0.0072	0.9949
**TFPAG-Net Full**	0.9886 ± 0.0058	0.9981
Time Branch Only	0.9458 ± 0.0260	0.9934
Freq Branch Only	0.9554 ± 0.0198	0.9953

**Table 7:** Ablation results of X-IIoTID.

Variant	Macro-F1 (Mean ± Sample SD)	Accuracy
TCN Only	0.9227 ± 0.0134	0.9937
Dual Branch	0.9301 ± 0.0298	0.9959
Dual Branch + Pyramid	0.9395 ± 0.0046	0.9955
**TFPAG-Net Full**	0.9466 ± 0.0290	0.9983
Time Branch Only	0.9187 ± 0.0200	0.9901
Freq Branch Only	0.9375 ± 0.00103	0.9947

**Table 8:** Ablation results of SWaT.

Variant	Macro-F1 (Mean ± Sample SD)	Accuracy
TCN Only	0.9356 ± 0.0077	0.9867
TCN + time	0.9505 ± 0.0032	0.9823
TCN + frequency	0.9448 ± 0.0048	0.9816
Dual Branch	0.9512 ± 0.0071	0.9828
Dual Branch + Pyramid	0.9519 ± 0.0069	0.9903
Pyramid + SE	0.9449 ± 0.0082	0.9841
Pyramid + PCMCI	0.9545 ± 0.0021	0.9847
**TFPAG-Net Full**	0.9607 ± 0.0059	0.9889

## Data Availability

The datasets used in this study are publicly available. The Edge-IIoTset dataset is available from IEEE DataPort at https://ieeexplore.ieee.org/document/9751703 (accessed on 10 May 2026). The X-IIoTID dataset is available from IEEE DataPort at: https://dx.doi.org/10.21227/mpb6-py55 (accessed on 10 May 2026). The SWaT dataset is available at https://www.sutd.edu.sg/itrust/swat (accessed on 18 May 2026).
